# Impact of Wnt/β-Catenin Inhibition on Cell Proliferation through *CDC25A* Downregulation in Soft Tissue Sarcomas

**DOI:** 10.3390/cancers12092556

**Published:** 2020-09-08

**Authors:** Esther Martinez-Font, Marina Pérez-Capó, Rafael Ramos, Irene Felipe, Carmen Garcías, Pablo Luna, Josefa Terrasa, Javier Martín-Broto, Oliver Vögler, Regina Alemany, Antònia Obrador-Hevia

**Affiliations:** 1 Group of Advanced Therapies and Biomarkers in Clinical Oncology, Health Research Institute of the Balearic Islands (IdISBa-IUNICS), Son Espases University Hospital, 07120 Palma, Spain; esther.martinez@ssib.es (E.M.-F.); marina.perez@ssib.es (M.P.-C.); mariad.garcias@ssib.es (C.G.); josefa.terrasa@ssib.es (J.T.); oliver.vogler@uib.es (O.V.); regina.alemany@uib.es (R.A.); 2 Medical Oncology Department, Son Espases University Hospital, 07120 Palma, Spain; pablo.luna@ssib.es; 3 Pathology Department, Son Espases University Hospital, 07120 Palma, Spain; rafaelf.ramos@ssib.es; 4 Epithelial Carcinogenesis Group, Spanish National Cancer Research Centre-CNIO, 28029 Madrid, Spain; ifelipe@cnio.es; 5 Medical Oncology Department, University Hospital Virgen del Rocío, 41013 Sevilla, Spain; jmartin@mustbesevilla.org; 6 Institute of Biomedicine of Sevilla, IBIS, HUVR, CSIC, Universidad de Sevilla, 41013 Sevilla, Spain; 7 Group of Clinical and Translational Research, Department of Biology, University of the Balearic Islands, 07122 Palma, Spain; 8 Molecular Diagnosis Unit, Son Espases University Hospital, 07120 Palma, Spain

**Keywords:** soft tissue sarcoma, Wnt signaling, β-catenin, CDC25A, PRI-724

## Abstract

**Simple Summary:**

Growing evidence suggests that Wnt signaling may be crucial for tumorigenesis and progression of soft tissue sarcomas (STS). Inhibitors of this pathway are currently in clinical trials or pre-clinical studies in order to validate its utility in different neoplasia. One of this inhibitors, PRI-724, is showing promising results for advanced pancreatic adenocarcinoma or ovarian cancer. We found that PRI-724 is able to suppress cell viability/proliferation and to increase cell death rates of soft tissue sarcomas cells in vitro. *CDC25A*, a target gene of Wnt signaling pathway, is essential for STS proliferation because its downregulation via siRNA was able to mimic the effect of PRT-724 on cell cycle arrest and evaluation of NCBI/GenBank data confirmed its overexpression in STS patients’ samples. Moreover, in vitro administration of PRI-724 along with standard STS chemotherapeutic drugs improved the efficacy of chemotherapy, suggesting that Wnt inhibition could be a promising new therapeutic strategy in STS.

**Abstract:**

The Wnt signaling pathway is an important cellular mechanism for regulating differentiation processes as well as cell cycle events, and different inhibitors of this pathway, for example, PRI-724, are showing promising results in clinical trials for treatment of advanced pancreatic adenocarcinoma or ovarian cancer. Growing evidence suggests that Wnt signaling may also be crucial for tumorigenesis and progression of soft tissue sarcomas (STS), a malignant neoplasm with few therapeutic options at an advanced state. Our study with several STS cell lines and primary cultures shows that inhibition of Wnt/β-catenin signaling with PRI-724 is able to suppress cell viability/proliferation and to increase cell death rates. TCF/β-catenin-mediated transcriptional activity is decreased in treated cells, leading to downregulation of its target genes *CCND1* and *CDC25A.* The latter was critical because its downregulation via siRNA was able to mimic the effect of PRI-724 on cell cycle arrest and cell death induction. An evaluation of NCBI/GenBank data confirmed that *CDC25A* mRNA is elevated in STS patients. Importantly, PRI-724 in combination with standard STS chemotherapeutics doxorubicin or trabectedin enhanced their antitumoral effect in a synergistic manner according to isobolographic analysis, suggesting that Wnt inhibition through PRI-724 could be a beneficial combination regime in patients with advanced STS.

## 1. Introduction

Soft tissue sarcomas (STS) are a heterogeneous group of malignant tumors of mesenchymal origin with 50 different histological subtypes. STS are part of the so-called rare cancers, since they have a low incidence on the population, representing only one per cent of all solid malignant neoplasia in adults [1,2]. Surgery with or without radiotherapy and chemotherapy is the first treatment option in patients with localized disease. Unfortunately, recurrence is frequent, reaching up to 60% or higher in high-risk localized populations. Therefore, systematic therapy for metastatic or unresectable patients is needed to offer some disease control [3]. The therapeutic options for inoperable or metastatic STS only offer a limited benefit on the prognosis and patients’ survival making the search for new molecular targets and the development of novel therapeutic approaches a still unmatched goal.

In many cancers, including soft tissue sarcomas, the Wnt/β-catenin signaling pathway is found to have aberrant activation [4,5,6]. Its normal function is to regulate the development and homeostasis of many tissues, including those derived from mesenchymal cell lineages. The canonical the Wnt/β-catenin signaling pathway is activated with the binding of extracellular Wnt ligand to Frizzled (FZD) receptors and low-density lipoprotein receptor-related proteins-5/6 (LRP5/6) co-receptors. This triggers an intracellular signaling cascade that inactivates the Axin-APC-GSK3β destruction complex, allowing the accumulation of non-phosphorylated β-catenin in the cytoplasm. Free non-phosphorylated β-catenin then translocates to the nucleus and binds to the T-cell factor/lymphoid enhancer factor-1 (TCF/LEF) family of transcription factors and coactivator complexes, thereby inducing transcription of Wnt target genes [7].

Wnt pathway is frequently dysregulated through a variety of genetic and epigenetic mechanisms, such as loss of function mutations in *APC* gene, especially in colorectal carcinomas, or point mutations in β-catenin (*CTNNB1*), which prevent its GSK3-dependent phosphorylation in a variety of tumors [5,6,8]. In the context of sarcomas, Wnt canonical pathway has been shown to be upregulated by other mechanisms [8,9]. It is known that SS18-SSX, the oncogenic fusion protein in synovial sarcoma, drives constitutively active β-catenin signaling allowing tumor formation [8,10,11]. Moreover, an increase in Wnt receptor expression or a loss of expression of secreted inhibitors due to increased β-catenin activity is frequently observed in osteosarcoma. Therefore, the aberrant Wnt/β-catenin signaling disrupts normal bone development [8,12]. On the other hand, we and others have described that the Wnt/β-catenin signaling pathway is constitutively activated in a panel of STS cell lines and tumor-derived cells (liposarcomas, leiomyosarcomas, synovial sarcomas and fibrosarcomas), which confer them a highly proliferative and survival capacity, with *CDC25A* being a key target gene [11,13,14].

Many efforts have been made over the past decades in order to target the canonical Wnt signaling in cancer. There are multiple Wnt/β-catenin signaling inhibitors, including biological and small molecules, which have shown promising activities in cancer therapy by disrupting the pathway at different points [15,16,17,18,19]. The biological compounds include antibodies, RNA interference molecules and recombinant proteins that target Wnt proteins and extracellular modulators of the pathway [19,20]. Small molecules can be divided into four categories according to their mechanism of action [19]. At present, the Class I types (e.g., PKF118-310, CGP049090, CWP232228) are receiving increasing attention because they block the final step of the whole signaling pathway, i.e., the interaction of β-catenin with TCF. As a result, they more efficiently bypass all aberrant activations of the Wnt signaling pathway caused by any upstream dysregulation [16,18,19,21,22,23]. Recently, our group showed that PKF118-310 effectively inhibits cell proliferation by inducing apoptosis in a panel of representative STS cell lines and primary cultures with upregulated Wnt/β-catenin signaling. Importantly, PKF118-310 simultaneously combined with doxorubicin, the standard first-line therapy in metastatic STS, increased its antitumoral effect in a synergistic manner [14].

**Figure 1:**
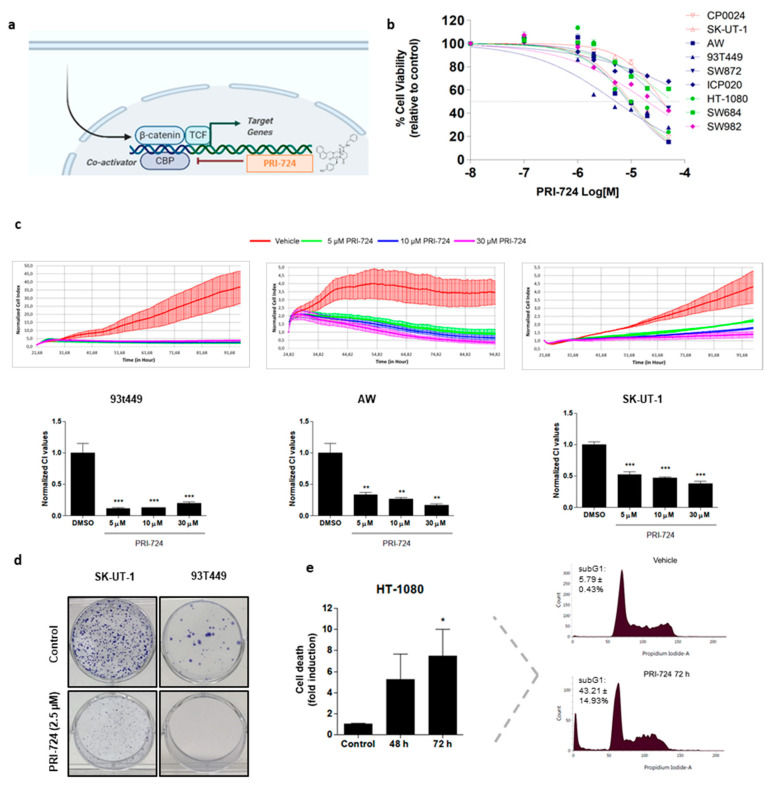
Inhibition of the interaction between β-catenin and its transcriptional coactivator CBP suppresses cell viability and colony formation of STS cell lines. (**a**) PRI-724 inhibits β-catenin interaction with its co-activator CBP. (**b**) STS cells were treated with PRI-724 (0.1–50 µM) and cell viability was determined after 48 h. Data is represented as mean ± SEM percentage of cell viability relative to vehicle-treated cells calculated from three independent experiments performed in triplicate. (**c**) STS cells were treated with PRI-724 (5, 10 and 30 µM) for 72 h and cell growth was continuously monitored using the RTCA MP Instrument. Cell Index (CI) values were normalized to the CI value at the time point of compound addition. (Upper panel) The CI profiles of PRI-724-treated cells and DMSO-treated cells (red line) reflect response to the respective treatments. Error bars show the standard deviation of the mean of triplicates of at least two independent experiments. (Lower panel) Quantification of Normalized CI values at 48 h. (**d**) STS cells were treated with PRI-724 (2.5 µM) every 96 h. After 9 days, colonies reached saturation and quantification of colony-forming ability was determined by counting the colonies using the ImageJ software (ImageJ 1.51j8, Wayne Rasband, NIH, USA). The panel shows representative results of at least three independent experiments. (**e**) Cells were treated with PRI-724 (10 µM) for 48 and 72 h, fixed with ethanol, stained with propidium iodide, and DNA content was determined by flow cytometry. Columns show the extent of cell death induction with the inhibitor represented as fold increase at 48 and 72 h. Each column represents mean ± SEM of three independent experiments. Histograms of vehicle-treated (control) and PRI-724-treated cells (10 µM) for 72 h are shown. The fluorescence values used to calculate the peaks corresponding to the sub-G1 phase (cell death) are indicated on each histogram. * *p* < 0.05, ** *p* < 0.01 and *** *p* < 0.001 compared with vehicle-treated cells.

Despite the fact that most of those Wnt inhibitors are in preclinical phases, a few of them, especially OMP-54F28 (Ipafricept) and PRI-724, are showing positive results in clinical trials for cancer treatment [15,16,21,22,23,24,25,26]. A phase I trial of Ipafricept in combination with chemotherapy in ovarian cancer reported that 35% of patients showed complete response, 47% exhibited a partial response, and 18% had stable disease [27]. PRI-724 belongs to the Class II small molecule Wnt inhibitors, which are antagonists of β-catenin transcriptional coactivators. PRI-724 specifically inhibits CBP (CREB-binding protein)/β-catenin interaction, thereby reducing the recruiting of β-catenin with its coactivator CBP [26,28] (Figure 1a). In a phase Ib study, PRI-724 exhibited modest antitumoral activity when combined with gemcitabine as second-line therapy in patients with advanced pancreatic adenocarcinoma [29]. In addition, a phase II clinical trial is currently underway in which PRI-724 is evaluated in combination with chemotherapy and bevacizumab for the treatment of patients with newly diagnosed metastatic colorectal cancer [30].

In the present work, we examined the antitumor efficacy of PRI-724 in a panel of prevalent STS cell lines and primary cultures. Our findings show that inhibition of CBP/β-catenin interaction suppresses the proliferation of a wide range of STS cell lines and patient-derived cells and promotes cell death, at least in part, via *CDC25A* downregulation. Furthermore, the combination of PRI-724 with conventional chemotherapeutic agents used in the treatment of metastatic STS, such as doxorubicin and trabectedin, increased their antitumoral effects in a synergistic manner, suggesting that CBP/β-catenin inhibitors, such as PRI-724, could also be a promising therapeutic option for STS patients, and should be further evaluated.

## 2. Results

### 2.1. Inhibition of CBP/β-Catenin Interaction Suppresses Cell Viability and Colony Formation of STS Cell Lines

To test the effect of CBP/β-catenin disruption on cell proliferation, STS cell lines and primary cultures representing different sarcoma histologies and mutational background were exposed to increasing concentrations of PRI-724 (0.1–50 µM) for 24, 48 and 72 h (Table S1). As shown in Figure 1b, after 48 h of treatment cell viability was reduced below 50% in 77.8% (7/9) of the cell lines tested. 93T449, AW, HT-1080 and CP0024 cell lines were more sensitive to treatment, with IC_50_ values ranging from 5.18 to 9.77 µM, compared with SW982, SW872 and SK-UT-1, with IC_50_ values ranging from 20.13 to 38.86 µM (Table S1 shows IC_50_ values for 48 h treatment). Two cell lines, the commercial fibrosarcoma cell line SW684 and the primary liposarcoma culture ICP020, were more resistant to this Wnt inhibitor, since it was not able to achieve a 50% inhibition of cell viability at any of the concentrations studied. In addition, similar results were obtained when cell growth was continuously monitored in liposarcoma AW, 93T449 and leiomyosarcoma SK-UT-1 cells under PRI-724 treatment by using the xCELLigence system (Figure 1c). To assess whether disruption of CBP/β-catenin interaction altered more phenotypic properties of tumoral cells, colony formation assay was performed in two cell lines with different sensitivity to PRI-724. After 9 days of treatment, PRI-724 (2.5 µM) significantly decreased the colony-forming ability of leiomyosarcoma SK-UT-1 and liposarcoma 93T449 cells. As shown in Figure 1d, the reduction effect of PRI-724 was 52.04 ± 4.12% in SK-UT-1 cells and 99.59 ± 0.22% in 93T449 cells, the latter being the most sensitive cell line to this Wnt inhibitor according to the proliferation assay. Taken together, PRI-724 was able to reduce viability as well as proliferation of STS cells, and no correlation between antitumoral effects and histology of cell lines could be established.

### 2.2. Inhibition of CBP/β-Catenin Interaction Promotes Cell Death of STS Cell Lines

Cell cycle profiling was performed on STS cells treated with PRI-724 by flow cytometry (Figure 1e and Figure S1). Treatment with PRI-724 for 48 and 72 h increased the sub-G1 phase of STS cells in a time-dependent manner, being statistically significant in only two of the five cell lines tested. At 10 µM, CBP/β-catenin inhibitor provoked in fibrosarcoma HT-1080 and leiomyosarcoma SK-UT-1 the highest effect on the sub-G1 phase, thus raising cell death in vehicle-treated cells after 72 h 8- and 4-fold, respectively. This effect of CBP/β-catenin inhibition was more moderate in the rest of the cell lines tested, increasing cell death 2-fold after 72 h of treatment. Taken together, these results indicate that PRI-724 exhibits antiproliferative activity, at least in part, by triggering cell death in STS cell lines. Additional studies performed with Annexin V/PI and assessment of the cleavage of PARP by immunoblot, as well as measurement of caspase 3/7 activities (Figure S2) ruled out that the involved mechanism of cell death provoked by PRI-724 treatment was apoptosis.

### 2.3. Inhibition of CBP/β-Catenin Interaction Reduces TCF/β-Catenin-Mediated Transcriptional Activity, but Not β-Catenin Subcellular Localization

**Figure 2:**
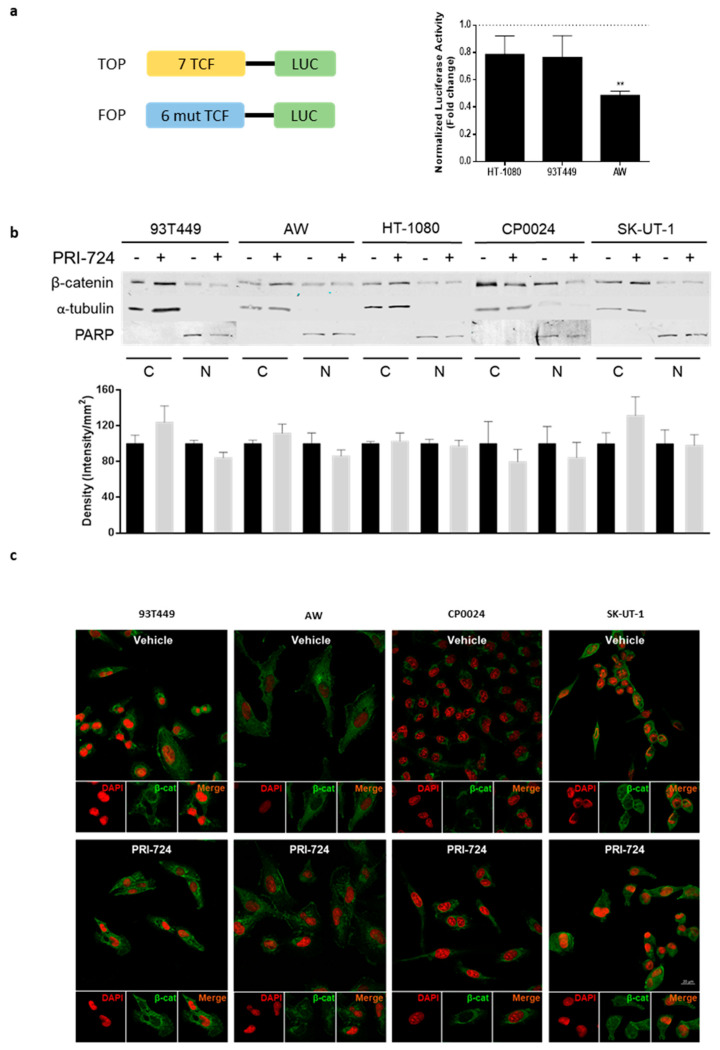
Inhibition of CBP/β-catenin interaction reduces TCF/β-catenin-mediated transcriptional activity, but not β-catenin subcellular localization. (**a**) STS cells were treated with PRI-724 (10 µM) or vehicle (control, DMSO) for 24 h and transfected with TCF reporter system plasmids. After 24 h of transfection, luciferase reporter activity was measured. Firefly luciferase was normalized to Renilla luciferase activity. Results are expressed as fold change of normalized luciferase activity values relative to those of each control. Each column represents mean ± SEM of two independent experiments performed in triplicate. ** *p* < 0.01 compared with vehicle-treated cells. (**b**) STS cells treated with PRI-724 (10 µM) for 48 h were subjected to subcellular fractionation. Upper panel shows the immunoreactive bands of β-catenin in cytoplasmic and nuclear fractions in a representative immunoblot of at least three independent experiments. α-Tubulin and PARP were used as a cytoplasmic and nuclear marker, respectively. Intensity quantification of the bands are shown in the bottom graphs. (**c**) STS cells were treated with PRI-724 (10 µM) for 48 h and fixed with methanol: acetone (1:1). β-catenin was visualized with the primary antibody β-catenin (D10A8) [(XP Rabbit mAb #8480, Cell Signaling Technology, Beverly, MA, USA)] followed by the addition of the secondary antibody Alexa Fluor 488 (Goat a-R A11008, Invitrogen S.A, Barcelona, Spain). DAPI was added to visualize the nuclei. Panel shows representative images taken with confocal microscope, ZEISS LSM 710 (Carl Zeiss Microscope GmbH, Jena, Germany), with a 40× objective and power of 512 × 512 pixels of at least two independent experiments performed in duplicate.

To further evaluate the effects of PRI-724 treatment on STS, we chose representative cell lines covering a wide range of sensitivity to this inhibitor, i.e., 93T449, AW, HT-1080, CP0024 and SK-UT-1 (Table S1). As we previously reported, STS cell lines have basal TCF/β-catenin mediated transcriptional activity [14] being similar to that of human colon cancer cells, whose Wnt pathway is known to be strongly activated. To evaluate the effect of CBP/β-catenin disruption on β-catenin transcriptional activity, TCF reporter activity was evaluated in STS cells treated with PRI-724 by TOP/FOPflash luciferase reporter assay. As shown in Figure 2a, PRI-724 treatment for 24 h led to a reduction of TCF/β-catenin reporter activity in fibrosarcoma HT-1080 cells and 93T449 and AW liposarcoma cells. The inhibitory effect of PRI-724 on TCF/β-catenin reporter activity was rather small in HT-1080 and 93T449 cells (20% of decrease), while it inhibited by 50% the β-catenin transcriptional activity in AW cells. Several studies have shown that compounds that disrupt the interaction between β-catenin and TCF/LEF transcription factors prevent nuclear accumulation of β-catenin [14,31]. To investigate whether the disruption of β-catenin interaction with CBP had a similar effect on β-catenin localization, SK-UT-1, HT-1080 and 93T449 with strong nuclear β-catenin staining, CP0024 with moderate nuclear staining of β-catenin and AW with weak β-catenin nuclear staining were treated with the PRI-724 at 10 µM for 48 h. After treatment, intracellular location of β-catenin was determined by nuclear/cytoplasmic fractionation and immunofluorescence showing that disruption of CBP/β-catenin interaction was not able to reduce nuclear β-catenin levels in the STS cell lines studied, instead a mild increase in nuclear β-catenin was detected by immunofluorescence in 93T449 cells (Figure 2b,c and Figures S3 and S4).

### 2.4. Inhibition of CBP/β-Catenin Interaction Reduces TCF/β-Catenin-Dependent Expression of Target Genes

**Figure 3:**
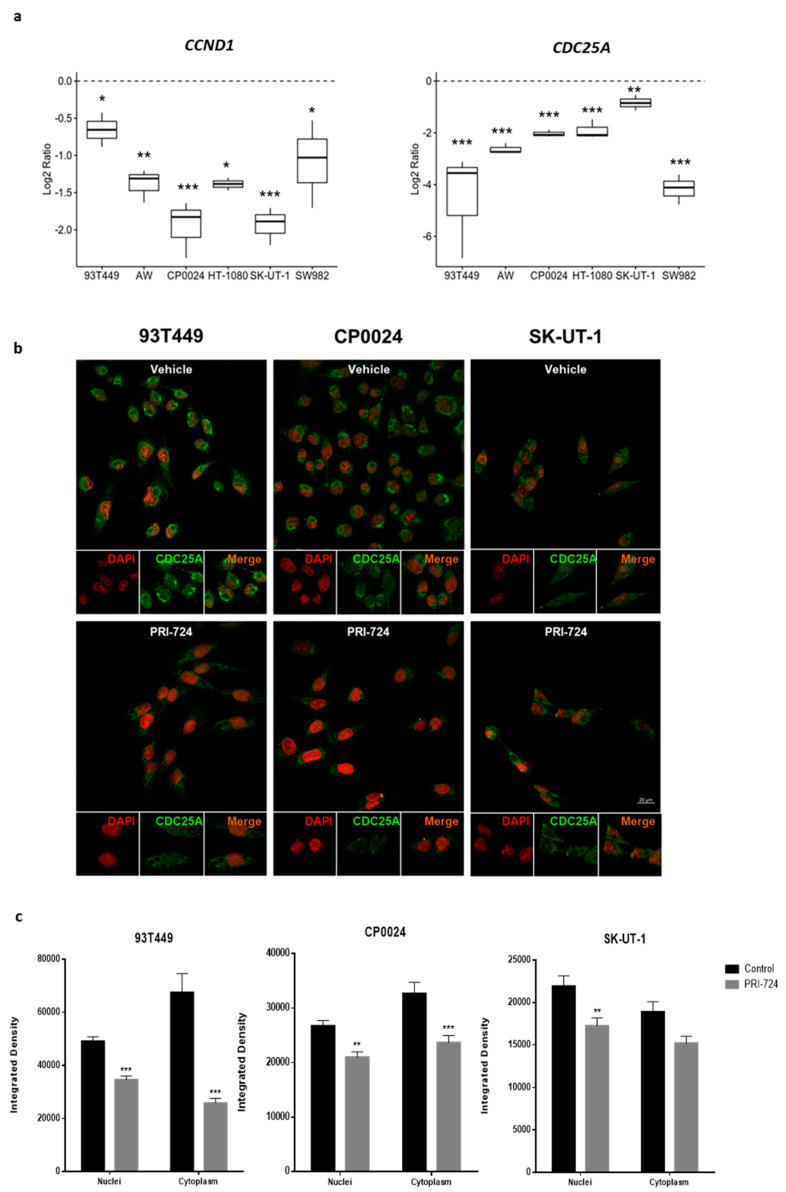
Inhibition of CBP/β-catenin interaction decreases the expression of TCF/β-catenin dependent target genes. (**a**) *CCND1* and *CDC25A* mRNA expression levels in STS cells treated with PRI-724 (10 µM) for 48 h. Each column represents means ± SEM of three independent determinations performed by duplicate. (**b**) STS cells were treated with PRI-724 (10 µM) for 48 h, fixed with methanol: acetone (1:1) and incubated with the primary antibody CDC25A (M-191) (sc-7157, Santa Cruz) followed by the addition of the secondary antibody Alexa Fluor 488 (Goat a-R A11008, Invitrogen). DAPI was added to visualize the nuclei. Panel shows representative images taken with confocal microscope, ZEISS LSM 710 (Carl Zeiss Microscope GmbH, Jena, Germany), with a 40× objective and power of 512 × 512 pixels of at least two independent experiments performed in duplicate. (**c**) Nuclear and cytoplasmic CDC25A protein levels were quantified measuring the integrated density by ImageJ software. Each column represents mean ± SEM of two independent experiments performed in duplicate. * *p* < 0.05, ** *p* < 0.01 and *** *p* < 0.001 compared with vehicle-treated cells.

To further investigate the effect of CBP/β-catenin disruption in STS cells, the expression of Wnt downstream signaling targets in STS treated with PRI-724 was assessed by RT-PCR. *CCND1* is a well-known cell cycle regulator and *CDC25A* has been described as a major regulator of cell cycle progression in STS [13,14], with both of them being TCF/β-catenin-dependent target genes. Previous results showed that *CDC25A* gene levels in STS cells are higher than those of hMSCs, a reference cell line with low levels of endogenous Wnt signaling [14]. Interestingly, in APC-mutated-SK-UT-1 cells, endogenous *CDC25A* levels were higher than those found in two colorectal cancer cell lines with strong intrinsic Wnt signaling activity used as positive controls. Treatment with PRI-274 revealed a progressive time-dependent downregulation of *CDC25A* gene in all cell lines tested, whereas *CCND1* gene expression decreased only during the first 48 h of treatment, recovering its expression afterwards (Figure 3a and Figure S5). As expected, the downregulation effect of PRI-724 on *CDC25A* expression was less pronounced in SK-UT-1 cells (reduction of 20%, 44% and 51% after 24, 48 and 72 h, respectively) (Table S1). To evaluate CDC25A protein levels, immunofluorescence analysis was performed due to inconsistent data obtained by immunoblot with different CDC25A antibodies. In line with the results of mRNA expression, they confirmed a significant reduction of CDC25A protein levels in STS cells after 48 h of PRI-724 treatment (10 µM). CDC25A protein was detected in cytoplasmic as well as nuclear compartments in the analyzed STS cells (Figure 3b,c). Liposarcoma 93T449 and leiomyosarcoma CP0024 cells, which were more sensitive to disruption of CBP/β-catenin interaction (Table S1), had higher levels of CDC25A protein in the cytoplasm than in the nuclei. Conversely, in leiomyosarcoma SK-UT-1 cells, one of the most resistant cell lines to this Wnt inhibitor (Table S1), the CDC25A expression was greater in the nucleus than in the cytoplasm. In agreement with their sensibility to PRI-724, the greatest inhibitory effect of this compound on CDC25A protein levels was observed in liposarcoma 93T449 cells (Figure 3b,c) when compared with leiomyosarcoma CP0024 or SK-UT-1 cells. Thus, PRI-724 exerted an inhibitory effect on expression of *CDC25A* being in line with the decrease of β-catenin transcriptional activity shown with the reporter assay.

### 2.5. CDC25A Is a Major Regulator of STS Cell Proliferation Mediated by β-Catenin Transcriptional Activity

**Figure 4:**
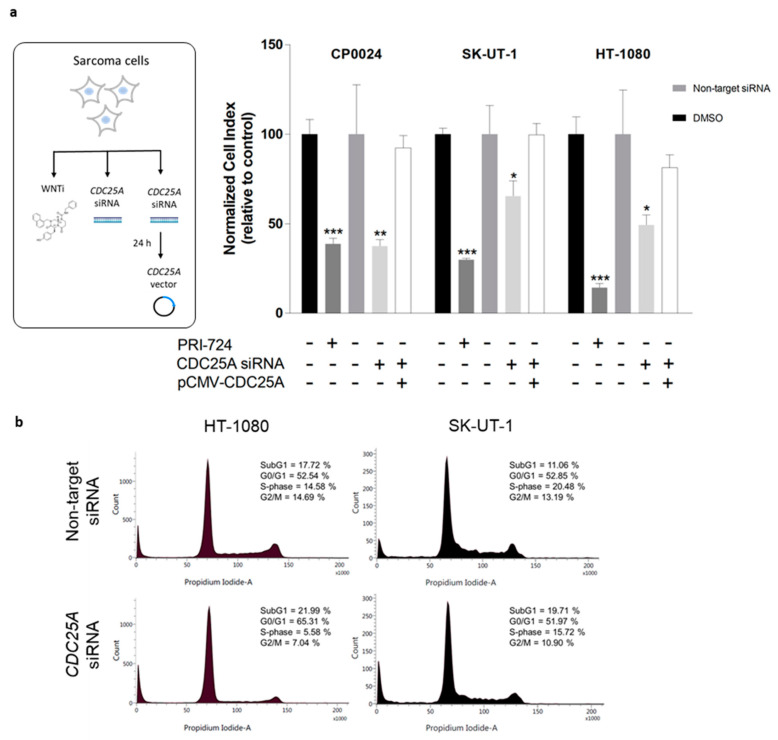
Depletion of *CDC25A* inhibits cell proliferation either via cell death induction or G1 arrest in STS cells. (**a**) STS cells were treated with PRI-724 (30 µM), transfected with *CDC25A* siRNA or Non-Target siRNA for 72 h. After 24 h of siRNA transfection, STS cells were co-transfected with pCMV-CDC25A plasmid or the empty vector pcDNA3.1 for 48 h. Cell growth was continuously monitored using the RTCA MP Instrument. Cell index (CI) values were normalized to each CI value at the time point of compound addition or transfection. Each column represents mean ± SEM of cell index values of two independent experiments performed in quadruplicate. (**b**) STS cells were transfected with *CDC25A* siRNA or Non-Target siRNA for 72 h, fixed in ethanol, stained with propidium iodide, and DNA content was determined by flow cytometry. Representative histograms of propidium iodide from the singlet gate are represented to display the phases of the cell cycle of at least three independent experiments. * *p* < 0.05, ** *p* < 0.01 and *** *p* < 0.001.

To elucidate the role of CDC25A in STS proliferation we used those STS cells lines with a higher expression of *CDC25A*, SK-UT-1, HT-1080 and CP0024, as determined in a previous work [14]. A small interfering RNA (siRNA) to specifically downregulate *CDC25A* gene expression was used (Figures S6 and S7). Fibrosarcoma HT-1080 and leiomyosarcoma CP0024 and SK-UT-1 cells were transiently transfected with *CDC25A* siRNA, and cell viability was continuously monitored using the xCELLigence system. As shown in Figure 4a, the inhibitory effect of PRI-724 at, 30 µM was evaluated, as this corresponded to the IC_50_ concentration regarding proliferation inhibition of the most resistant cell line SK-UT-1. In fact, cell proliferation of CP0024 decreased by 66%, which was very similar to the 55% reduction induced by the downregulation (silencing) of *CDC25A* expression. However, *CDC25A* downregulation caused a moderate reduction of cell proliferation in SK-UT-1 and HT-1080 cell lines (22% and 49%, respectively) compared to that caused by CBP/β-catenin inhibition (70% and 89%, respectively). Interestingly, exogenous expression of *CDC25A* by co-transfecting cells with pCMV-CDC25A 24 h after siRNA transfection rescued cell index values after 48 h in all cells tested (Figure 4a white column). As CDC25A is an important regulator of cell cycle progression, we performed cell cycle analysis by flow cytometry to determinate the effect of *CDC25A* gene downregulation in the different phases of the cell cycle. As seen in Figure 4b, *CDC25A* downregulation inhibited cell proliferation in HT-1080 and SK-UT-1 cells by impairing cell cycle progression through the G1 checkpoint or by cell death induction. These results show that CDC25A is a relevant mediator of proliferation in STS and its downregulation leads to a decrease of cell growth as treatment with PRI-724.

**Figure 5:**
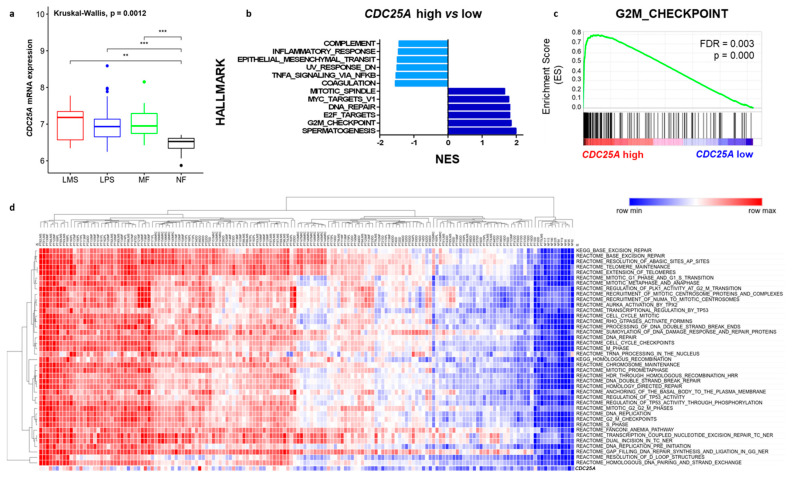
Overexpression of *CDC25A* mRNA levels in STS is related to cell cycle and DNA repair pathways enrichment. Raw data was collected from the National Center for Biotechnology Information (NCBI)/GenBank GEO web site (GSE21122) and normalized in R using Bioconductor and associated packages. (**a**) For each box plot, median and ranges are indicated. ** *p* < 0.01 and *** *p* < 0.001. LMS: Leiomyosarcoma, LPS: Liposarcoma, MF: Myxofibrosarcoma and NF: normal fat. (**b**) Gene Set Enrichment Analysis (GSEA) table summarizing the enrichment in gene signatures (hallmarks) in *CDC25A* high versus low STS patient samples. Samples were separated in two groups: *CDC25A* high and *CDC25A* low (based on Q1/Q4 distribution) and GSEA was used to analyze gene signatures and pathways enrichment. Enrichment Score (ES) reflects the degree to which a gene set is overrepresented at the top or bottom of a ranked list of genes. The normalized enrichment score (NES) is the primary statistic for examining gene set enrichment results and can be used to compare analysis results across gene sets. Gene sets were selected by their statistical significance filtered by FDR q-value < 0.25 and Nom *p*-value < 0.05. (**c**) Snapshots of G2M Checkpoint gene set significantly enriched in *CDC25A* high STS patient samples. (**d**) Heatmap of top 40 enriched Canonical pathways gene sets derived from the KEGG and Reactome pathways databases from MSigDB in STS samples determined by single-sample Gene Set Enrichment Analysis (ssGSEA). Each ssGSEA enrichment score represents the degree to which the genes in a particular gene set are coordinately up- or down-regulated within a sample.

To further address the clinical relevance of *CDC25A* in STS, its mRNA expression levels were examined in a broad series of patient samples (*n* = 149) gathered from a public TCGA data set [32] including 26 leiomyosarcomas, 89 liposarcomas, 34 myxofibrosarcomas and 9 control adipose tissue samples. As shown in Figure 5a *CDC25A* mRNA expression was similar between STS subtypes and higher when compared with control normal fat tissue samples. In conclusion, as shown by our results and public data, *CDC25A* is overexpressed in STS samples and seems to be involved in tumor proliferation. To further investigate this question, we analyzed the tumor same sample set by using Gene Set Enrichment Analysis (GSEA) in order to identify curated gene signatures whose expression changed significantly in *CDC25A* high STS patient samples. For that purpose, samples were separated in two groups, *CDC25A* high and *CDC25A* low, based on *CDC25A* first and last quartile of expression. As shown in Figure 5b,c and Table S3, gene signatures related with cell cycle regulation and DNA repair were significant enriched in the *CDC25A* high subgroup. Single-sample GSEA (ssGSEA) enrichment score represents the degree to which the genes in a particular gene set are coordinately up- or down-regulated within a sample. This score characterizes cell state in terms of the activity levels of biological processes and pathways rather than through the expression levels of individual genes. The ssGSEA performed on the entire sample revealed an up-down regulation of those pathways enriched in *CDC25A* high tumor subgroup. As shown in Figure 5d, samples nicely separate in two clusters (tumor and normal tissue) accompanied by a concordance with *CDC25A* expression levels. The samples with highest *CDC25A* levels showed higher scores in cell cycle and DNA repair related processes.

### 2.6. Inhibition of CBP/β-Catenin Interaction Enhances Antitumoral Effect of Standard Chemotherapeutic Agents in STS Cells

**Figure 6:**
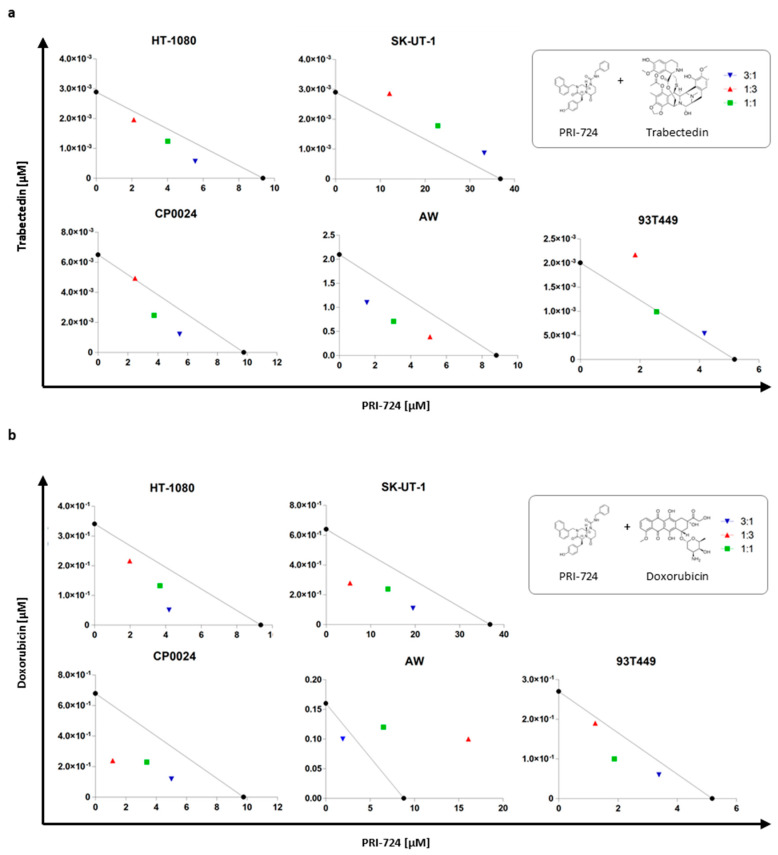
PRI-724 enhances antitumoral effect of trabectedin and doxorubicin in STS cells. STS cells were treated with fixed dose ratio combinations of PRI-724 and trabectedin (**a**) or doxorubicin (**b**) for 48 h. Isobolograms were constructed by plotting the IC_50_ of trabectedin (**a**) or doxorubicin (**b**) on the y-axis and the IC_50_ of PRI-724 on the x-axis, being the line that connects these two points the additivity line. Combination index (CI) was calculated using data from three independent experiments performed in triplicate.

Combination of the CBP/β-catenin inhibitor PRI-724 with trabectedin or doxorubicin at different fixed dose ratio combinations, led to an enhanced reduction of STS cell viability, achieving synergistic effects (CI < 1) in most of cell lines tested after 48 h of treatment as seen in Table S2 and Figure 6. One of the combinations that exerted an antagonist effect was with PRI-724 and trabectedin in APC-mutated SK-UT-1 cell line.

## 3. Discussion

In a previous work, we demonstrated that the Wnt signaling pathway is constitutively activated in a broad range of STS cell lines and primary cultures independently of the sarcoma subtype [14]. In this study, we explore the antitumoral effects of Wnt pathway inhibition on STS cell lines and patient-derived primary cultures using the small molecule PRI-724, which blocks CBP/β-catenin interaction and is currently in clinical trials for different cancer types but not for STS.

PRI-724 was able to strongly reduce cell proliferation in vitro by inducing nonapoptotic cell death or by impairing cell cycle progression through the G1 checkpoint (Figure 1). This effect was less pronounced in cell lines with mutations in the *APC* gene (SK-UT-1 and SW684), and this is probably related to the elevated activation of the pathway as a consequence of this mutation, which is not completely reverted by PRI-724. Our results are in agreement with Ma and colleagues, who found that ICG-001, a first generation of CBP/β-catenin inhibitor, is able to reduce viability in colorectal carcinoma cells SW480 [33]. Moreover, as pointed by Emami and colleagues, who found the same effects of ICG-001 on cell viability using SW480 and HCT116 cells [34], most probably PRI-724 would be cytostatic at lower concentrations and cytotoxic, increasing caspase activity, at higher concentrations. Accordingly, in our study, caspase activity decreased upon treatment with PRI-724 (10 µM), which would be consistent with an inability to induce apoptotic processes due to cell cycle arrest.

At the molecular level, PRI-724 treatment caused attenuation of TCF/β-catenin mediated transcriptional activity in STS cells, and it was not accompanied by a change of the subcellular localization of β-catenin (Figure 2), whereas disruption of TCF/β-catenin interaction by PKF118-310 provokes destabilization of β-catenin and reduction of its nuclear localization as our previous study indicated [14,31]. However, it has to be taken into account that both compounds interact with the Wnt pathway through different molecular mechanisms. Once in the nucleus, β-catenin must recruit one of the two transcriptional coactivators CBP (cAMP-response-element-binding protein (CREB)-binding protein) or p300 (E1A-binding protein) to induce transcription of Wnt target genes. For several years, the functions of these two coactivators have been described as redundant due to their high degree of homology. Despite this, emerging studies [35,36,37,38] indicate that CBP and p300 are not interchangeable, and it has been reported that each of these two coactivators is responsible for a distinct transcriptional program guiding the cell to either proliferate or retain potency in the case of CBP or to initiate differentiation in case of p300 [33,37,39,40]. In this context, PKF118-310 blocks the binding of β-catenin to the TCF/LEF complex, thereby decreasing the amount of bound β-catenin in the nucleus, whereas PRI-724 selectively blocks the CBP/β-catenin interaction without interfering with the interaction of β-catenin with p300 [34]. This fact would explain that, although the inhibitory effect of PRI-724 on total TCF/β-catenin-mediated transcriptional activity was mild and not statistically significant in HT-1080 and 93T449 cells (Figure 2a), PRI-724 strongly reduced their proliferation (Figure 1; Table S1) most probably by inhibiting the expression of TCF/CBP/β-catenin dependent target genes which are related to cell proliferation, as is *CDC25A* (Figure 3). As PRI-724 leaves the TCF/LEF binding site unaltered, β-catenin can still bind to TCF/LEF and recruit transcriptional cofactors other than CBP, thereby avoiding a decrease of nuclear β-catenin localization. Such a differential regulation of TCF/LEF activity has also been observed in the case of the formerly mentioned CBP/β-catenin antagonist ICG-001. This compound induces apoptosis through downregulation of the protein survivin, whose expression is as well only increased by the transcriptional coactivator CBP but not p300 [33]. Altogether, our results point to a cellular model with a nonredundant function of CBP and p300, which seem to be able to trigger a different set of transcriptional routes.

As a result of the attenuation of CBP/β-catenin mediated transcriptional activity, PRI-724 significantly reduced the expression of TCF/β-catenin dependent target genes related with cell proliferation such as *CCND1*, a cell cycle regulator well-known by its role in tumorigenesis [41,42], and *CDC25A*. The reduction of CDC25A expression at mRNA and protein level is of special clinical relevance, since CDC25A has been described as a major cell proliferation mediator in STS, both in vitro and in vivo [13]. We observed that, in general terms, *CDC25A* was more strongly downregulated by PRI-724 than *CCND1* (Figure 3a). However, in SK-UT-1 cells, which were rather resistant to the antiproliferative effect of PRI-724, downregulation of *CDC25A* levels were also rather weak. In contrast, in 93T449 cells a drastic downregulation of *CDC25A* was paralleled by an only weak downregulation of *CCND1*, and these cells were highly sensitive to PRI-724. Accordingly, depletion of *CDC25A* by a specific siRNA significantly decreased STS proliferation through cell death induction or G1 arrest (Figure 4). Additionally, in this work CDC25A protein was found in both nuclear and cytoplasmic compartments in all analyzed STS cells and, interestingly, those STS cell lines in which CBP/β-catenin inhibition by PRI-724 most effectively inhibited cell proliferation were the ones with higher levels of CDC25A in cytoplasm (Figure 3b). In fact, the extent of CDC25A protein reduction in cytoplasm after treatment with PRI-724 determined the extent of sensitivity to this Wnt inhibitor (e.g., 93T449 cells) (Figure 3b). These results are in line with previously published data indicating that cytoplasmic localization of CDC25A is related to anti-apoptotic and pro-survival effects on ovarian and skin tumor cells [43,44], and therefore leads to a dependency of tumor progression on CDC25A. Taken together, the fact that inhibition of CBP/β-catenin interaction by PRI-724 suppressed cell proliferation to a similar extent to that provoked by *CDC25A* downregulation further strengthens the role of *CDC25A* as a major regulator of STS cell proliferation and its importance as pivotal molecular target of this compound.

High *CDC25A* mRNA levels have been reported previously by us and others in a wide range of representative Wnt-activated STS cell lines [13,14]. In parallel, Bertucci and colleagues reported a positive correlation between Wnt activation and expression of *CDC25A* mRNA in a large series of sarcoma patient samples [45]. The results of available public data analysis, comparing STS patient samples with normal tissues and evaluating gene signatures and pathways enriched in *CDC25A* high STS patient samples confirmed these results (Figure 5) and highlight that this gene target is actually of clinical relevance in STS independently of STS subtype.

Although the Wnt signaling pathway was discovered over 30 years ago, it was not before the last decade that drug development focused on this pathway as a new therapeutic approach in oncology. However, clinical Wnt modulator candidates must take into account the different roles of the pathway, which maintain the fine balance between cell proliferation versus differentiation. Therefore, only few specific therapeutic agents targeting this pathway have entered clinical trials until today. On the other hand, a growing body of evidence indicates that a combination therapy with such compounds may be more effective than monotherapy in most malignancies [26,28,29,30]. For this reason, several clinical trials are currently testing PRI-724 in combination with conventional chemotherapeutics. Our work demonstrates that combination of PRI-724 with trabectedin or doxorubicin, both standard chemotherapeutic agents in advanced STS, may also have a beneficial clinical effect. In fact, the combination of PRI-724 with either of these drugs increased their antitumor activity in a synergistic manner in most of the STS cells studied. Interestingly, it has been reported that CBP/β-catenin inhibitors are able to improve the chemotherapy-associated toxicity in preclinical models [46].

## 4. Materials and Methods

### 4.1. Cell Lines, Primary Cell Culture and Reagents

93T449 cell line was established at Hospital de l’Archet, Nice, Francia (2012) [47,48], and kindly provided by Dr. Florence Pedeutour (UMR 6549, CNRS UNSA, Université de Nice-Sophia Antipolis, France). Pharma Mar S.A. (2011) kindly donated cell lines SW684 and SW872 (available from the American Type Culture Collection, ATCC). HT-1080, SK-UT-1 and SW982 cell lines were purchased from the ATCC (Manassas, VA, USA) (2011). AW cell line was established at CNIO (2011) [49], and kindly provided by Dr. Amancio Carnero. 93T449 cell line was maintained in RPMI 1640 medium (PAA, Piscataway, NJ, USA) supplemented with 1% L-glutamine (Invitrogen S.A, Barcelona, Spain), 10% FBS (PAA), 100 units/mL penicillin/streptomycin (PAA), 1% Ultroser (Pall Life Sciences, Port Washington, NY, USA) and 0.5% Fungizone (Invitrogen). SW872, SW684 and SW982 were maintained in Leibovitz’s medium with L-glutamine supplemented with 10% FBS, 100 units/mL penicillin/streptomycin and 1 mM HEPES (Sigma-Aldrich, Madrid, Spain). HT-1080 was maintained in MEM liquid with Earle’s Salts medium with L-glutamine (PAA) supplemented with 10% FBS, 100 units/mL penicillin/streptomycin and 1 mM HEPES. AW was maintained in F-10 Ham medium (Gibco) supplemented with 1% Ultroser, 10% FBS, 100 units/mL penicillin/streptomycin and 1mM HEPES. SK-UT-1 was maintained in DMEM High glucose with L-glutamine (PAA) supplemented with 10% FBS, 100 units/mL penicillin/streptomycin, 1 mM HEPES and 1% of sodium pyruvate (Sigma-Aldrich) and MEM non-essential amino acid solution (Sigma-Aldrich). Two primary cell cultures (CP0024 (2012) and ICP020 (2015)) were derived from a resected leiomyosarcoma and liposarcoma. The biopsies were minced in culture medium and then disaggregated by 30 min incubation with collagenase (Gibco, 221 U/mg). Cells were cultured in RPMI 1640 medium supplemented with 20% FBS, 100 units/mL penicillin/streptomycin. Informed consent was obtained from all patients in accordance with the guidelines of the Ethical Committee of Clinical Investigation (CEI-IB, Palma, Spain) and the Declaration of Helsinki. Cells were grown in a humidified incubator containing 5% CO_2_ at 37 °C.

Wnt inhibitor, PRI-724, was purchased from Selleck Chemicals LLC (Houston, TX, USA). Doxorubicin was kindly provided by the Pharmacology Department of Son Espases University Hospital. Trabectedin was provided by Pharmamar (Madrid, Spain).

### 4.2. Cell Viability Assay (MTT)

The methylthiazoletetrazolium (MTT) method was used to measure cell viability using the CellTiter 96^®^AQueous One Solution (Promega, Madison, WI, USA) following the manufacturer’s instructions. Briefly, 5000 cells were seeded in 96-well plates for 24 h and exposed to increasing concentrations of the Wnt inhibitor for 24, 48 and 72 h followed by addition of CellTiter reagent. Absorbance at 490 nm was detected using a multi-well scanning spectrophotometer (Synergy H1 microplate reader, Bio-tek, Winooski, VT, USA). The mean percentage of cell viability relative to vehicle-treated cells was estimated, the compound concentration resulting in 50% inhibition of cell viability (IC50) was determined using GraphPad software (GraphPad Software Inc., San Diego, CA, USA).

### 4.3. Real-Time Cell Analysis (RTCA)

Cells were seeded in 96-well E-plates with integral sensor electrode arrays (ACEA Biosciences, San Diego, CA, USA). After 24 h, the cells were treated with different concentrations of the Wnt inhibitor and cell proliferation was monitored noninvasively, in real time and label-free using the xCELLigence Real-Time Cell Analyzer (ACEA Biosciences) for up to 96 h. The 96-well E-plates used with the xCELLigence system have gold electrodes on their bottom surface, serving as sensors of alternating current modified by the number of adherent cells and cell status (including cell viability, morphology, and adhesion). A dimensionless parameter termed cell index (CI) was derived as a relative change in measured electrical impedance to represent cell status. CI normalized to the time of medium switch/treatment was presented in xCELLigence recordings.

### 4.4. Colony-Forming Assay

Cellular ability to grow and form colonies was evaluated by clonogenic assay. Briefly, 500 cells were seeded in 6-well plates and treated with PRI-724 (2.5 µM) for 9 days (medium was changed every 96 h). Once colonies reached saturation cells were fixed with cold methanol, washed with PBS and stained with crystal violet 1% (Sigma-Aldrich, Madrid, Spain) for 20 min. Cellular colony-forming ability was quantified using the ImageJ software.

### 4.5. Cell Cycle Analysis

Flow cytometry was performed to analyze the effect of Wnt inhibitor on cell cycle. Briefly, 30,000 cells were seeded in 24-well plates. After 24, 48 and 72 h of treatment, cells were washed in PBS and fixed in 90% ethanol, collected by centrifugation and stained in 500 μL of a mixture of propidium iodide (50 µg/mL) (Sigma-Aldrich, Madrid, Spain) and ribonuclease A (50 µg/mL) (Sigma-Aldrich, Madrid, Spain) diluted in PBS for 1 h. Cell populations at different stages of the cell cycle were estimated based on their DNA content in flow cytometer using the BD system Verse BD FACScan (Coulter Epics XL-MSL; Beckman Coulter, Fullerton, CA, USA) and software FACSuit (BD Bioscience, Franklin Lakes, NJ, USA). Cellular apoptosis was studied with Annexin V/PI (Molecular Probes™ Dead Cell Apoptosis Kit with Annexin V Alexa Fluor™ 488 & Propidium Iodide (PI), ThermoFisher V13241, Waltham, MA, USA) for flow cytometry, measurement of caspases 3 and 7 activities by means of the luminometric Caspase-Glo 3/7 Assay, (Promega G8091, Madison, WI, USA), and finally with the assessment of the cleavage of PARP by immunoblot analysis (#9542S, Cell Signaling Technology).

### 4.6. Western Blotting and Antibodies

Cells were seeded in 6-well plates, and after 48 h of treatment with vehicle or Wnt inhibitor, nuclear and cytoplasmic fractions were obtained using Nuclear Extract kit (Active Motif) and following the manufacturer’s instructions. The antibodies used were the following: β-catenin (#8480, Cell Signaling Technology, 1:1000 dilution), PARP (#9542S, Cell Signaling Technology, 1:1000 dilution) and α-tubulin (#T9026, Sigma-Aldrich, 1:1000 dilution). The secondary antibodies were: Donkey anti-Rabbit (E 365D5) and Donkey anti-Mouse (E 510KC) (LI-COR, 1:10,000 dilution). Western blotting was performed, subjecting 10 µg of each lysed fraction to SDS-PAGE, transferring onto a PVDF membrane (Immobilon-FL, Merck Millipore, Cork, Ireland) and incubating with the indicated antibodies. Immunoreactivity intensity of the bands was analyzed with the Odyssey imaging system from LI-COR and quantified by Quantity One 1-D Analysis Software (BIO-RAD, Hercules, CA, USA).

### 4.7. Immunofluorescence

Cells were grown in a Lab-Tek II Chamber Slide (Lab-Tek) chamber, and 48 h after treatment with vehicle or Wnt inhibitor cells were washed with PBS plus 0.2% BSA and fixed with methanol: acetone (1:1), blocked with a 5% BSA solution, incubated with the primary antibody β-Catenin (D10A8) (#8480, Cell Signaling Technology, 1:250 dilution), and CDC25A (M-191) (sc-7157, Santa Cruz, 1:250 dilution) and with the secondary antibody (Alexa Fluor 488 Goat a-R A11008, Invitrogen, 1:150 dilution). DAPI (Fisher Scientific) was used to visualize the nuclei. Five images per condition of each individual experiment were taken using the confocal microscope, ZEISS LSM 710, with a 40X objective and power of 512 × 512 pixels. The ImageJ software was used to quantify β-catenin and CDC25A staining intensity. Briefly, using the DAPI channel, the nuclei of the cells in each field of view were selected manually and the software automatically measured the integrated density of selected areas.

### 4.8. mRNA Expression Analysis

Total RNA was isolated using Trizol Plus RNA Purification Kit (Ambion) according to the manufacturer’s instructions. 300 ng of RNA were reverse-transcribed into cDNA using High-Capacity cDNA Reverse Transcription Kit (Applied Biosystems). Subsequent real-time PCR reactions were performed using the TaqMan probes method (CFX96 Real-Time System, C1000 Thermal Cycler, BIO-RAD). Relative gene expression was normalized to β-2-microglobulin using the 2-∆∆Ct method [50]. The TaqMan probes used were the following: β-2-microglobulin (Hs99999907), CDC25A (Hs00947994) and CCND1 (Hs00765553) (Applied Biosystems).

### 4.9. TCF Reporter Assay, Small Interfering RNA (siRNAs), Plasmids and Transfections

Transient transfection was performed using the Lipofectamine Plus (Invitrogen) method. TCF reporter system plasmids (pTOPFLASH and pFOPFLASH) and the empty vector pcDNA3.1 were kindly donated by Dr. Gabriel Capellà, expression plasmid pCMV-CDC25A (HG11291-M-N) was purchased from Abyntek Biopharma, S.L (Bizkaia, Spain). Luciferase reporter assay was performed using the Dual-Luciferase Reporter Assay System (Promega) according to the manufacturer’s instructions. Luciferase activity was quantified by multi-well scanning spectrophotometer (Synergy H1 microplate reader, Bio-tek) 24 h after transfection and treatment with Wnt inhibitor. Firefly luciferase activity was normalized to the corresponding Renilla luciferase activity.

*CDC25A* gene was silenced by reverse transfection with the ON-TARGETplus Human *CDC25A* (993) siRNA-SMARTpool (Thermo Scientific Dharmacon, Waltham, MA, USA), which contains four different *CDC25A* siRNAs. Lipofectamine^®^RNAiMAX Reagent (Invitrogen) was used as transfection reagent following the manufacturer’s instructions. The Non-target (D-001810-10-05) pool (Thermo Scientific Dharmacon) was used as control. Briefly, transfection complexes were prepared by adding 5 µL (6-well plate) or 0.18 µL (96-well plate) of Lipofectamine^®^RNAiMAX Reagent, 10 nM of *CDC25A* siRNA-SMARTpool in 750 µL (6-well plate) or 30 µL (96-well plate) of Opti-MEMTM (ThermoFisher Scientific). During the 30 min of incubation, cells were trypsinized and suspended in complete growth medium without antibiotics to a concentration of 50,000 cell/mL (6-well plate) or 13,500 cell/mL (96-well plate).

Cells seeded with added transfection complex were incubated for 24 h or 72 h at 37 °C in a humidified incubator containing 5% CO_2_.

### 4.10. Data Collection and Analysis

Raw data from Barretina et al. 2010 public data set (GSE21122) was collected from the National Center for Biotechnology Information (NCBI)/GenBank GEO web site. After quality control, raw data was normalized by RMA [51] in R using Bioconductor and related packages.

Gene Set Enrichment Analysis (GSEA) was performed based on *CDC25A* first and last quartile of expression in STS tumor samples using the GSEA 4.0.3 software (www.broadinstitute.org/gsea). Gene sets were obtainged from H (Hallmark gene sets) and C2 (Curated gene sets) collections from MSigDB (Molecular Signature Database) [52,53]. Significant enriched signatures were identified using FDR *q*-value < 0.25 and nominal *p*-value < 0.05.

Single-sample GSEA (ssGSEA) was performed using the GenePattern web-tool (v3.9.11_b270) with Canonical pathways gene sets derive from KEGG and Reactome databases from MSigDB (https://cloud.genepattern.org/GenePattern) [52,54].

### 4.11. Statistical Analysis

The antiproliferative effect of the combination of Wnt inhibitor with doxorubicin and trabectedin was assessed by Loewe’s isobolographic analysis (1927, 1928, 1953), which considered three types of interactions: additivity (CI = 1), synergy (CI < 1) and antagonism (CI > 1). Each combination was evaluated by cell viability assay to determine the 50% inhibition of cell viability (IC_50_) value. By plotting the IC_50_ value of doxorubicin or trabectedin on the Y-axis and the IC_50_ value of PRI-724 on the X-axis combination isobolograms were obtained. The line connecting these two points it is known as line of additivity.

Results are expressed as mean ± SEM from n independent experiments performed in duplicate or triplicate. Statistical evaluations were assessed by GraphPadPrism, GraphPad Software, Inc. To detect the difference of quantitative values between the different groups, an ANOVA (parametric), along with a Bonferroni test for multiple comparisons, or Kruskal–Wallis (non-parametric) were used. Differences were considered statistically significant at *p* < 0.05 and are indicated by: *** *p* < 0.001, ** *p* < 0.01, and * *p* < 0.05.

## 5. Conclusions

**Figure 7:**
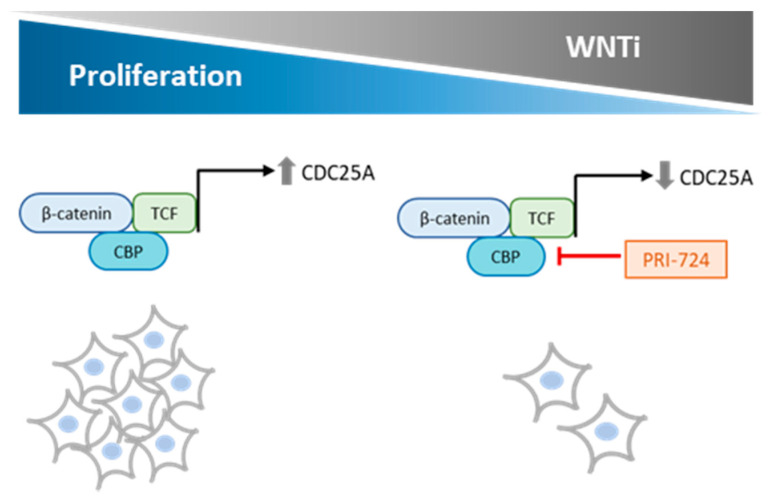
Proposed model for PRI-724 reduction of STS cell proliferation. PRI-724 binds to CBP, leading to inactivation of TCF/CBP/β-catenin-dependent target gene transcription, such as *CDC25A*, a major player in STS cell proliferation.

In summary, our study provides evidence that the Wnt inhibitor PRI-724 reduces STS cell proliferation in vitro by inducing cell death or G1 arrest, at least in part, through *CDC25A* downregulation. Moreover, these results are highly translational because they demonstrate that inhibition of *CDC25A*, which is highly expressed in a broad series of patient samples, could represent a potential new therapeutic drug target (Figure 7). Moreover, concomitant treatment with the Wnt inhibitor PRI-724 and trabectedin or doxorubicin is able to enhance the chemotherapeutic effect of standard STS drugs. This new strategy could benefit sarcoma patients with molecular alterations in the Wnt signaling pathway, especially if CDC25A deregulation is observed.

## Supplementary Materials

The following are available online at https://www.mdpi.com/2072-6694/12/9/2556/s1, Figure S1: Inhibition of CBP/β-catenin interaction promotes cell death in STS cells, Figure S2: Determination of cellular apoptosis upon treatment with PRI-724, Figure S3: Inhibition of CBP/β-catenin interaction does not reduce β-catenin subcellular localization, Figure S4: Inhibition of CBP/β-catenin interaction does not alter β-catenin nuclear levels in STS cells, Figure S5: Inhibition of CBP/β-catenin interaction decreases the expression of TCF/β-catenin dependent target genes, Figure S6: *CDC25A* mRNA expression levels in STS cells after transfection of *CDC25A* siRNA for 48, 72 and 96 h, Figure S7: *CDC25A* mRNA expression levels in STS cells, Table S1: STS cell lines characteristics and IC_50_ values for PRI-724 after 48 h, Table S2: Combination Index (CI) values for combinations of the CBP/β-catenin inhibitor with standard chemotherapeutic agents: trabectedin and doxorubicin in STS cells, Table S3: Significant top 100 pathways enriched in *CDC25A* high STS samples.
