# Supplementary material for: Impact of Wnt/β-Catenin Inhibition on Cell Proliferation through CDC25A Downregulation in Soft Tissue Sarcomas
Source: Cancers (Basel). 2020 Sep 8;12(9):2556. doi: 10.3390/cancers12092556 (PMC7564873; doi:10.3390/cancers12092556)
Supplement: Supplementary file 1 [file cancers-12-02556-s001.zip › cancers-908685-suppl-final.pdf]

cancers

MDPI

Article

Impact of Wnt/β-Catenin Inhibition on Cell Proliferation through *CDC25A* Downregulation in Soft Tissue Sarcomas

Esther Martinez-Font, Marina Pérez-Capó, Rafael Ramos, Irene Felipe, Carmen Garcías, Pablo Luna, Josefa Terrasa, Javier Martín-Broto, Oliver Vögler, Regina Alemany and Antònia Obrador-Hevia

Supplementary Figures

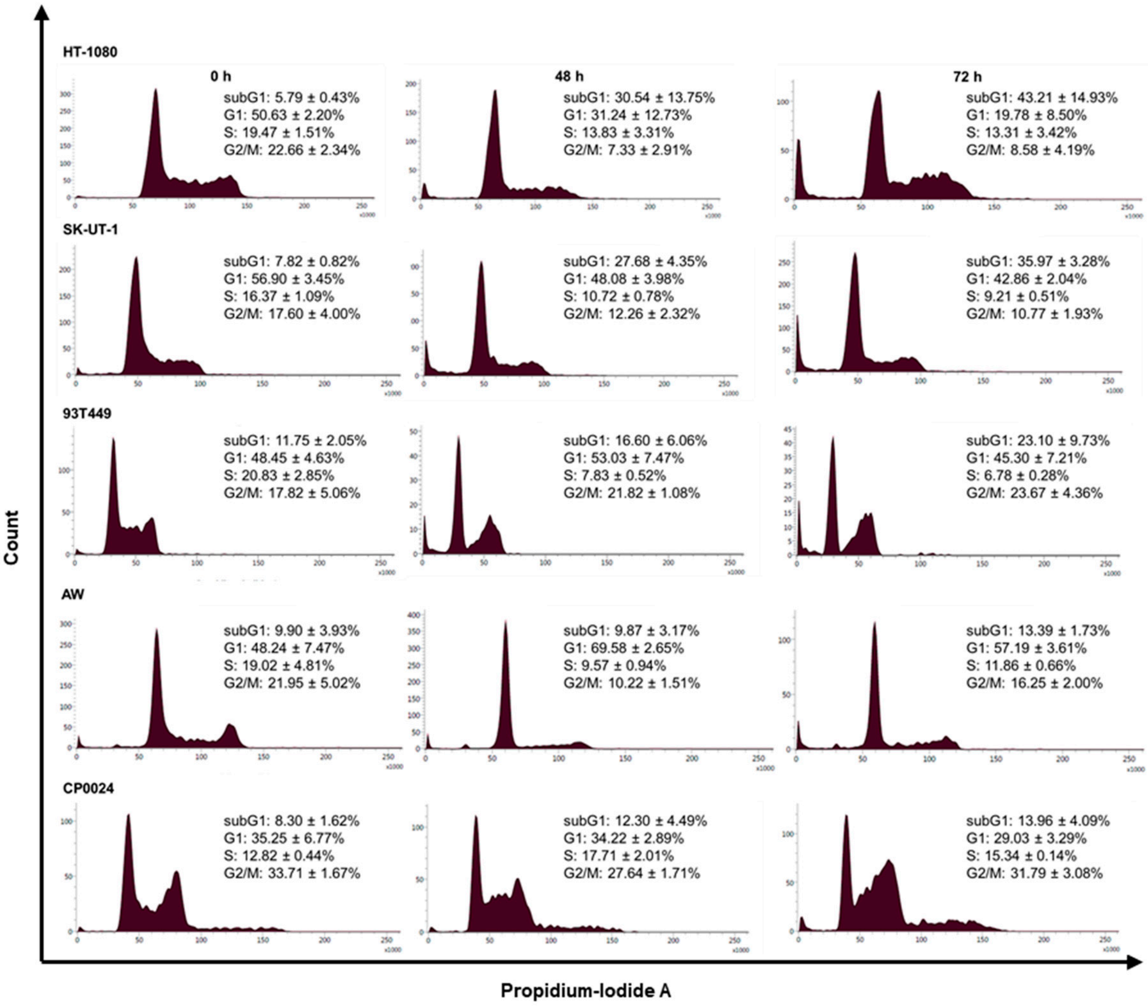

Figure S1. Inhibition of CBP/β-catenin interaction promotes cell death in STS cells. Cells were treated with PRI-724 (10 μM), fixed with ethanol, stained with propidium iodide, and DNA content was determined by flow cytometry. Histograms of PRI-724-treated cells for 0, 48 and 72 h are shown. The fluorescence values (count) used to calculate the peaks corresponding to the sub-G1, G1 and the G2/S/M phase are indicated on each histogram.

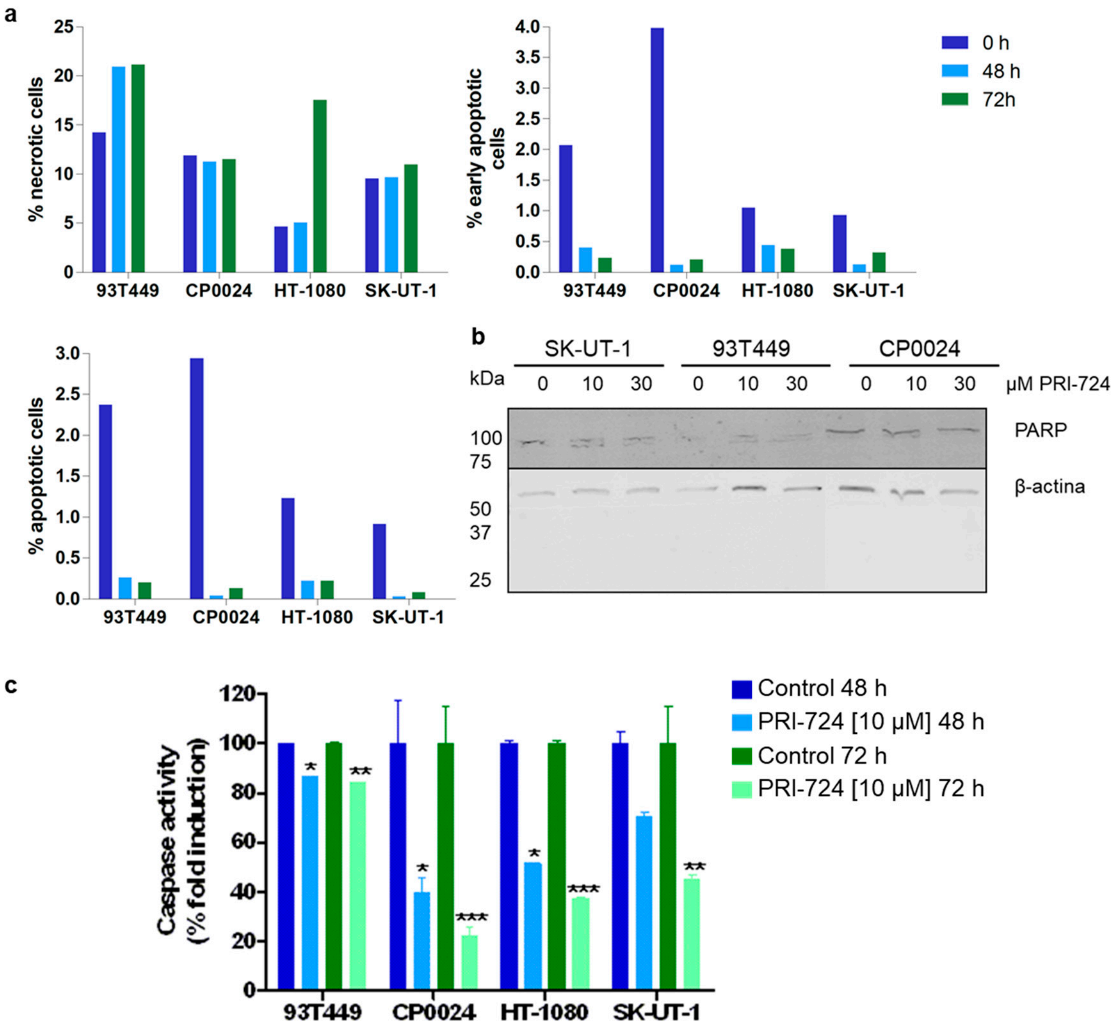

Figure S2. Determination of cellular apoptosis upon treatment with PRI-724 a) Detection of apoptosis by concurrent staining with Annexin V and PI. STS cells were treated with PRI-724 (10 µM) for 48 and 72 h. Cells were subsequently stained with Annexin V and PI and their fluorescence was measured by flow cytometry. Columns show the percentage of necrotic cells, and cells at early or late stage of apoptosis. b) STS cells were treated with PRI-724 (10 µM) for 48 and 72 h. Panel show the immunoreactive bands of PARP and β-actin, used as loading control. c) Caspase 3/7 activation upon treatment with PRI-724. STS cells were treated with PRI-724 (at 10 µM) and incubated for 48 and 72 h. Caspase activity was determined using the Caspase-Glo 3/7 Assay Kit. Caspase activity is represented as fold induction relative to vehicle-treated cells, and data represents mean ± S.E.M. from two independent determinations performed in triplicate.*, $p < 0.05$; **, $p < 0.01$ and ***, $p < 0.001$ compared with vehicle-treated cells.

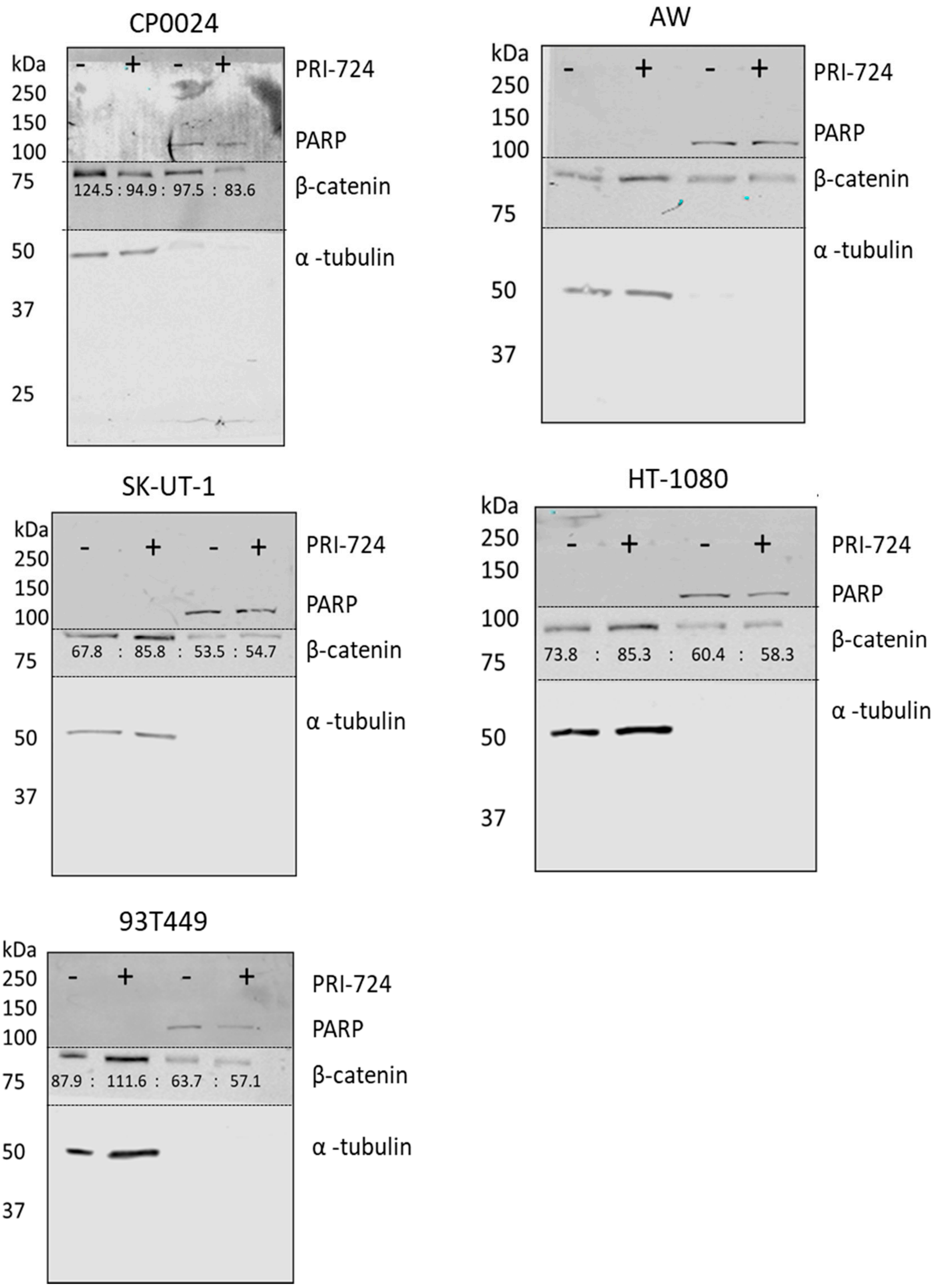

Figure S3. Inhibition of CBP/β-catenin interaction does not alter β-catenin subcellular localization. STS cells treated with PRI-724 (10 μM) for 48 h were subjected to subcellular fractionation. Upper panel show the immunoreactive bands of β-catenin (#8480, Cell Signaling, 92 kDa) in cytoplasmic and nuclear fractions in a representative immunoblot of at least three independent experiments. α-Tubulin (#T9026, Sigma-Aldrich, 50 kDa) and PARP (#9542S, Cell Signaling, 116 kDa) were used as a cytoplasmic and nuclear marker, respectively.

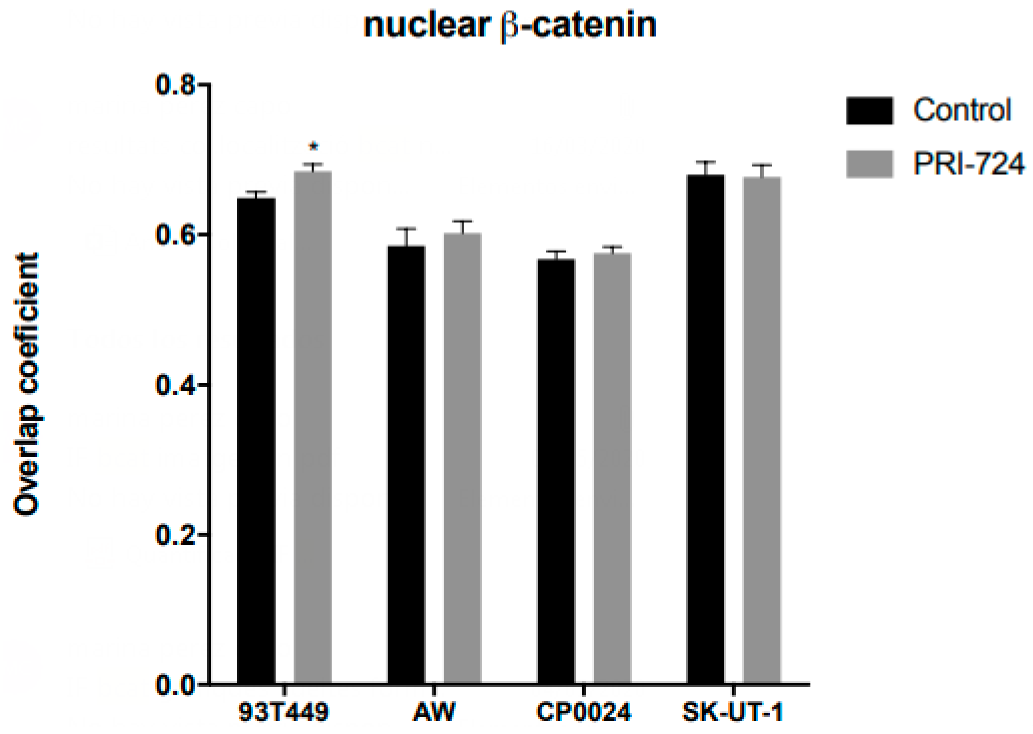

Figure S4. Inhibition of CBP/β-catenin interaction does not alter β-catenin nuclear levels in STS cells. 48h after treatment with PRI-724 (10 μM), cells were fixed with methanol: acetone (1:1) and incubated with the primary antibody β-catenin (D10A8) [(XP Rabbit mAb #8480, Cell Signaling Technology)] followed by the addition of the secondary antibody Alexa Fluor 488 (Goat a-R A11008, Invitrogen). DAPI was added to visualize the nuclei. Images were taken with confocal microscope (ZEISS LSM 710, with a 40X objective and power of 512 × 512 pixels) and colocalization analysis was made using ZEN 2011 software to detect the presence of β-catenin in the nucleus. PRI-724 treated cells presented similar protein levels of nuclear β-catenin to vehicle-treated cells. Each column represents means ± SEM of at least two independent experiments performed by duplicate. *, $p < 0.05$–compared with vehicle-treated cells.

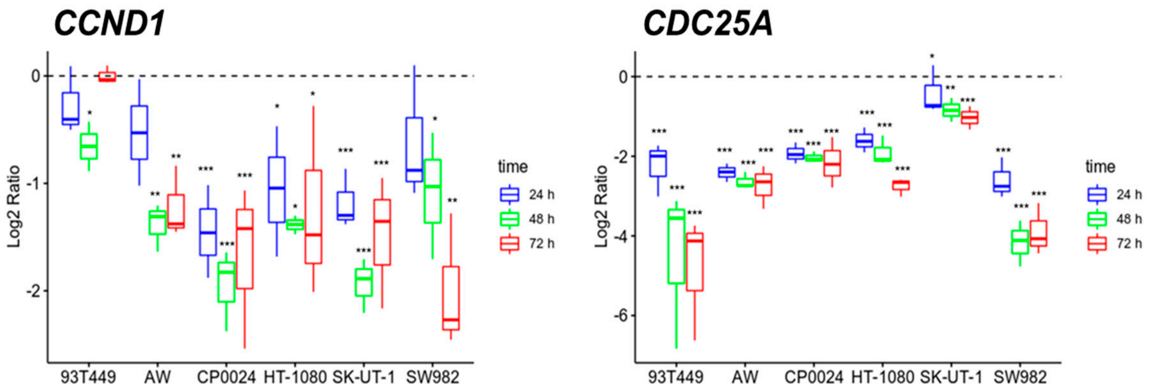

Figure S5. Inhibition of CBP/β-catenin interaction decreases the expression of TCF/β-catenin dependent target genes. *CCND1* and *CDC25A* mRNA expression levels in STS cells treated with PRI-724 (10 μM) for 24, 48 and 72 h. Each column represents means ± SEM of three independent determinations performed by duplicate. *, $p < 0.05$, **, $p < 0.01$ and ***, $p < 0.001$ compared with vehicle-treated cells.

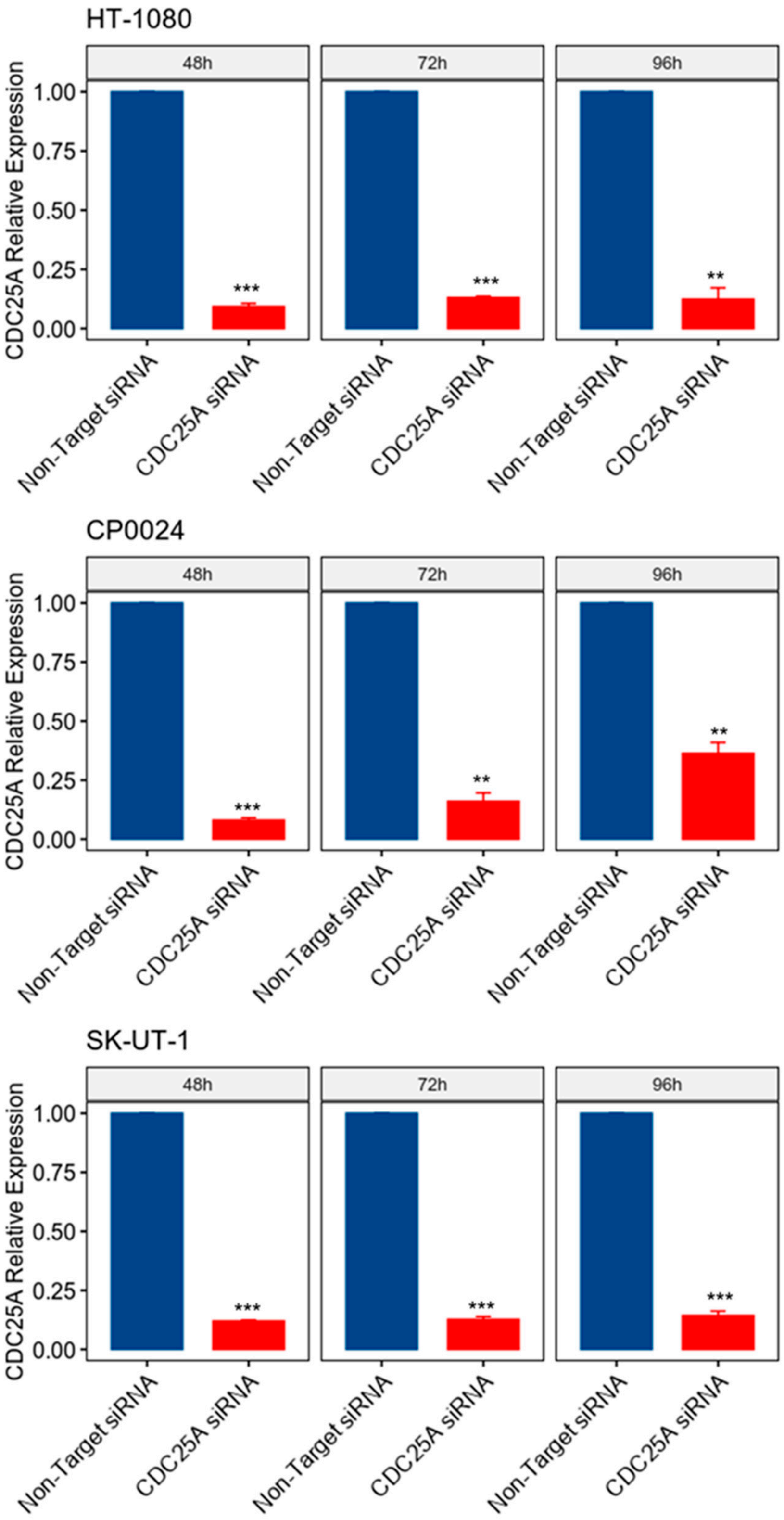

Figure S6. *CDC25A* mRNA expression levels in STS cells after transfection of *CDC25A* siRNA for 48, 72 and 96 h. Each column represents means ± SEM of three independent determinations performed by duplicate. **, $p < 0.01$ and ***, $p < 0.001$ compared with non-target siRNA transfected cells.

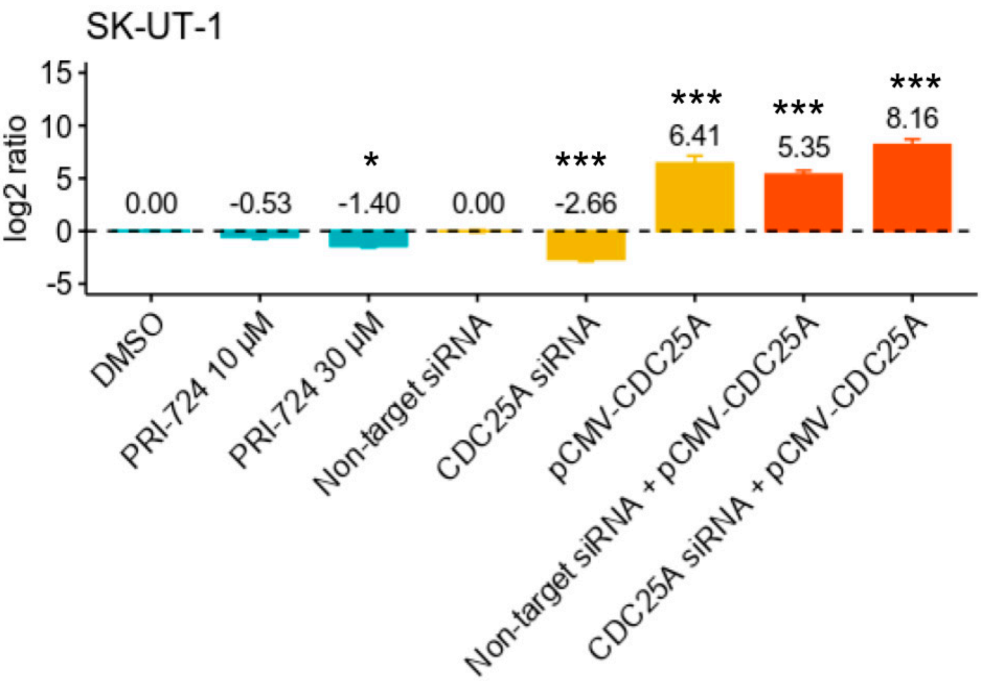

Figure S7. *CDC25A* mRNA expression levels in STS cells. SK-UT-1 cells were treated with PRI-724 (10 and 30 μM), transfected with CDC25A siRNA or non-target siRNA for 72 h. After 24 h of siRNA transfection, cells were co-transfected with pCMV-CDC25A plasmid or the empty vector pcDNA3.1 for 48 h. Each column represents mean ± SEM of three independent experiments performed in duplicate. *, $p < 0.05$ and ***, $p < 0.001$ compared with their respective control cells.

Supplementary Tables

Table S1. STS cell lines characteristics and IC_{50} values for PRI-724 after 48 h.

Cell line	Histology	Mutations		Other mutated genes	PRI-724 IC_{50} value (μM)
		APC	β-catenin (*CTNNB1*)		
**93T449**	**Liposarcoma**	**NA**	NA	*MDM2, CDK4, HMGA2*	5.18 ±0.95
**AW**	Liposarcoma	NA	NA	NA	8.80 ±0.99
**HT-1080**	Fibrosarcoma	No	No	*CDKN2A, IDH1, NRAS*	9.35 ±1.25
**CP0024**	Leiomyosarcoma	NA	NA	NA	9.77 ±1.01
**SW982**	Synovial Sarcoma	No	No	*BRAF, CDKN2A*	20.13 ±3.21
**SW872**	Liposarcoma	No	No	*BRAF, CDKN2A, PTEN, TP53*	36.03 ±5.15
**SK-UT-1**	Leiomyosarcoma	(c.4661delA) (c.3286C>T)	No	*PIK3A, PTEN, RB1, TP53*	38.86 ±4.31
**SW684**	Fibrosarcoma	(c.5460C>T)	(c.1213C>T)	*TP53*	>50
**ICP020**	Liposarcoma	NA	NA	NA	>50

NA: not available Data actualized from [14].

Table S2. Combination Index (CI) values for combinations of the CBP/β-catenin inhibitor with standard chemotherapeutic agents: trabectedin and doxorubicin in STS cells.

	PRI-724:Trabectedin			PRI-724:Doxorubicin		
**Cell line**	**1:1**	**1:3**	**3:1**	**1:1**	**1:3**	**3:1**
93T449	0.99	1.44	1.07	0.73	0.96	0.87
AW	0.68	0.77	0.70	1.48	2.44	0.87
HT-1080	0.86	0.90	0.79	0.78	0.85	0.60
CP0024	0.77	1.01	0.75	0.69	0.47	0.69
SK-UT-1	1.23	1.20	1.31	0.75	0.58	0.71

Table S3. Significant top 100 pathways enriched in *CDC25A* high STS samples. Green: DNA repair related pathways, Blue: Cell cycle pathways.

C2: curated gene sets enriched in *CDC25A* high vs low					
CDC25A high FDR q-val < 0.25 NOM p-val < 0.05	SIZE	ES	NES	NOM p-val	FDR q-val
REACTOME_RECRUITMENT_OF_NUMA_TO_MITOTIC_CENTROSOMES	80	0,68	2,05	0,000	0,014
REACTOME_DNA_DOUBLE_STRAND_BREAK_REPAIR	116	0,71	2,03	0,000	0,005
KEGG_HOMOLOGOUS_RECOMBINATION	27	0,77	2,03	0,000	0,005
REACTOME_AURKA_ACTIVATION_BY_TPX2	65	0,73	2,03	0,000	0,005
REACTOME_HOMOLOGY_DIRECTED_REPAIR	89	0,73	2,01	0,000	0,006
REACTOME_REGULATION_OF_TP53_ACTIVITY	140	0,60	2,00	0,000	0,006
REACTOME_DNA_REPAIR	242	0,63	1,99	0,000	0,006
KEGG_BASE_EXCISION_REPAIR	30	0,78	1,99	0,000	0,006
REACTOME_REGULATION_OF_TP53_ACTIVITY_THROUGH_PHOSPHORYLATION	82	0,65	1,98	0,002	0,006
REACTOME_ANCHORING_OF_THE_BASAL_BODY_TO_THE_PLASMA_MEMBRANE	83	0,61	1,97	0,000	0,006
REACTOME_RECRUITMENT_OF_MITOTIC_CENTROSOME_PROTEINS_AND_COMPLEXES	69	0,69	1,97	0,000	0,006
REACTOME_CELL_CYCLE_MITOTIC	435	0,70	1,95	0,000	0,007
REACTOME_HDR_THROUGH_HOMOLOGOUS_RECOMBINATION_HRR	55	0,77	1,94	0,000	0,007
REACTOME_REGULATION_OF_PLK1_ACTIVITY_AT_G2_M_TRANSITION	79	0,67	1,94	0,000	0,007
REACTOME_DNA_REPLICATION	115	0,76	1,93	0,000	0,007
REACTOME_MITOTIC_PROMETAPHASE	172	0,72	1,93	0,000	0,007
REACTOME_MITOTIC_G2_G2_M_PHASES	174	0,63	1,93	0,000	0,007
REACTOME_PROCESSING_OF_DNA_DOUBLE_STRAND_BREAK_ENDS	57	0,72	1,92	0,002	0,007
REACTOME_BASE_EXCISION_REPAIR	41	0,77	1,92	0,000	0,007
REACTOME_TELOMERE_MAINTENANCE	39	0,74	1,91	0,000	0,007
REACTOME_EXTENSION_OF_TELOMERES	39	0,74	1,91	0,000	0,007
REACTOME_RESOLUTION_OF_ABASIC_SITES_AP_SITES	34	0,81	1,91	0,000	0,007
REACTOME_CELL_CYCLE_CHECKPOINTS	224	0,71	1,91	0,000	0,007
REACTOME_G2_M_CHECKPOINTS	122	0,71	1,91	0,002	0,007
REACTOME_RESOLUTION_OF_D_LOOP_STRUCTURES	27	0,77	1,90	0,000	0,007
REACTOME_M_PHASE	303	0,64	1,90	0,000	0,007
REACTOME_FANCONI_ANEMIA_PATHWAY	26	0,74	1,90	0,000	0,007
REACTOME_CHROMOSOME_MAINTENANCE	60	0,77	1,89	0,000	0,008
REACTOME_TRANSCRIPTION_COUPLED_NUCLEOTIDE_EXCISION_REPAIR_TC_NER	72	0,63	1,89	0,000	0,008
REACTOME_MITOTIC_METAPHASE_AND_ANAPHASE	198	0,66	1,88	0,002	0,008
REACTOME_MITOTIC_G1_PHASE_AND_G1_S_TRANSITION	140	0,71	1,88	0,000	0,008
REACTOME_HOMOLOGOUS_DNA_PAIRING_AND_STRAND_EXCHANGE	38	0,79	1,88	0,000	0,008
REACTOME_TRNA_PROCESSING_IN_THE_NUCLEUS	50	0,62	1,88	0,002	0,008
REACTOME_DNA_REPLICATION_PRE_INITIATION	79	0,73	1,88	0,002	0,008
REACTOME_S_PHASE	142	0,68	1,88	0,002	0,008
REACTOME_DUAL_INCISION_IN_TC_NER	59	0,64	1,88	0,000	0,008
REACTOME_RHO_GTPASES_ACTIVATE_FORMINS	117	0,67	1,88	0,000	0,008
REACTOME_GAP_FILLING_DNA_REPAIR_SYNTHESIS_AND_LIGATION_IN_GG_NER	23	0,85	1,88	0,000	0,008
REACTOME_TRANSCRIPTIONAL_REGULATION_BY_TP53	303	0,53	1,87	0,000	0,007
REACTOME_SUMOYLATION_OF_DNA_DAMAGE_RESPONSE_AND_REPAIR_PROTEINS	68	0,60	1,87	0,000	0,007
REACTOME_RESOLUTION_OF_D_LOOP_STRUCTURES_THROUGH_SYNTHESIS_DEPENDENT_STRAND_ANNEALING_SDSA	25	0,78	1,87	0,000	0,007
REACTOME_G2_M_DNA_DAMAGE_CHECKPOINT	54	0,74	1,87	0,000	0,008
REACTOME_NONHOMOLOGOUS_END_JOINING_NHEJ	32	0,66	1,86	0,002	0,008
REACTOME_DUAL_INCISION_IN_GG_NER	38	0,70	1,86	0,002	0,008
KEGG_CELL_CYCLE	114	0,72	1,85	0,000	0,008
REACTOME_RECOGNITION_OF_DNA_DAMAGE_BY_PCNA_CONTAINING_REPLICATION_COMPLEX	28	0,81	1,85	0,000	0,008
REACTOME_MITOTIC_SPINDLE_CHECKPOINT	91	0,72	1,85	0,000	0,009
REACTOME_NUCLEAR_ENVELOPE_NE_REASSEMBLY	64	0,65	1,84	0,004	0,009
KEGG_PROGESTERONE_MEDIATED_OOCYTE_MATURATION	73	0,61	1,84	0,002	0,009
REACTOME_SEPARATION_OF_SISTER_CHROMATIDS	160	0,64	1,84	0,004	0,009
REACTOME_KINESINS	44	0,79	1,84	0,000	0,009
REACTOME_TRANSLESION_SYNTHESIS_BY_Y_FAMILY_DNA_POLYMERASES_BYPASSES_LESIONS_ON_DNA_TEMPLATE	33	0,74	1,84	0,002	0,009
KEGG_MISMATCH_REPAIR	22	0,86	1,83	0,000	0,009
REACTOME_MEIOTIC_RECOMBINATION	24	0,65	1,83	0,002	0,009
REACTOME_SUMOYLATION_OF_DNA_REPLICATION_PROTEINS	43	0,70	1,83	0,000	0,009
REACTOME_TRANSCRIPTIONAL_REGULATION_BY_E2F6	30	0,75	1,83	0,000	0,009
REACTOME_RESOLUTION_OF_SISTER_CHROMATID_COHESION	106	0,73	1,83	0,000	0,009
REACTOME_DNA_DAMAGE_BYPASS	41	0,73	1,82	0,002	0,009
REACTOME_HDR_THROUGH_SINGLE_STRAND_ANNEALING_SSA	33	0,75	1,82	0,000	0,010
REACTOME_SUMOYLATION_OF_RNA_BINDING_PROTEINS	42	0,60	1,82	0,000	0,010
REACTOME_TERMINATION_OF_TRANSLESION_DNA_SYNTHESIS	28	0,79	1,82	0,000	0,010
REACTOME_NUCLEOTIDE_EXCISION_REPAIR	99	0,58	1,81	0,002	0,010
REACTOME_GENE_SILENCING_BY_RNA	61	0,58	1,81	0,002	0,010
REACTOME_INTERACTIONS_OF_VPR_WITH_HOST_CELLULAR_PROTEINS	35	0,68	1,81	0,000	0,010
REACTOME_PROCESSING_OF_CAPPED_INTRON_CONTAINING_PRE_MRNA	198	0,55	1,80	0,009	0,010
REACTOME_ACTIVATION_OF_ATR_IN_RESPONSE_TO_REPLICATION_STRESS	34	0,87	1,80	0,000	0,010
REACTOME_POLYMERASE_SWITCHING_ON_THE_C_STRAND_OF_THE_TELOMERE	15	0,91	1,80	0,000	0,011
KEGG_PYRIMIDINE_METABOLISM	75	0,59	1,80	0,000	0,011
KEGG_DNA_REPLICATION	34	0,87	1,79	0,000	0,011
REACTOME_TRANSPORT_OF_MATURE_TRANSCRIPT_TO_CYTOPLASM	65	0,57	1,79	0,002	0,011
REACTOME_TRANSCRIPTION_OF_THE_HIV_GENOME	64	0,53	1,79	0,008	0,011
REACTOME_SNRNP_ASSEMBLY	47	0,63	1,79	0,002	0,011
KEGG_CYSTEINE_AND_METHIONINE_METABOLISM	32	0,62	1,79	0,000	0,012
KEGG_OOCYTE_MEIOSIS	92	0,63	1,79	0,002	0,011
REACTOME_TRNA_PROCESSING	87	0,53	1,78	0,004	0,011
REACTOME_HCMV_EARLY_EVENTS	68	0,60	1,78	0,000	0,012
KEGG_NUCLEOTIDE_EXCISION_REPAIR	42	0,68	1,77	0,002	0,013
REACTOME_ACTIVATION_OF_THE_PRE_REPLICATIVE_COMPLEX	31	0,88	1,77	0,000	0,013
REACTOME_TELOMERE_C_STRAND_LAGGING_STRAND_SYNTHESIS	25	0,83	1,77	0,000	0,013
REACTOME_VIRAL_MESSENGER_RNA_SYNTHESIS	42	0,62	1,77	0,002	0,013
REACTOME_MRNA_SPLICING	154	0,54	1,76	0,022	0,013
REACTOME_NUCLEAR_ENVELOPE_BREAKDOWN	47	0,69	1,76	0,002	0,014
REACTOME_TRANSCRIPTIONAL_REGULATION_BY_SMALL_RNAS	43	0,61	1,76	0,004	0,014
REACTOME_SWITCHING_OF_ORIGINS_TO_A_POST_REPLICATIVE_STATE	79	0,65	1,75	0,007	0,014
REACTOME_HIV_ELONGATION_ARREST_AND_RECOVERY	31	0,59	1,75	0,007	0,015
REACTOME_REPRODUCTION	64	0,56	1,75	0,002	0,015
REACTOME_RNA_POLYMERASE_II_TRANSCRIPTION_TERMINATION	49	0,57	1,75	0,011	0,015
REACTOME_RESOLUTION_OF_AP_SITES_VIA_THE_MULTIPLE_NUCLEOTIDE_PATCH_REPLACEMENT_PATHWAY	23	0,84	1,75	0,000	0,015
REACTOME_ASSEMBLY_OF_THE_PRE_REPLICATIVE_COMPLEX	63	0,65	1,75	0,009	0,015
REACTOME_HIV_TRANSCRIPTION_INITIATION	43	0,55	1,75	0,006	0,015
REACTOME_G0_AND_EARLY_G1	24	0,83	1,74	0,000	0,015
REACTOME_SUMOYLATION_OF_UBIQUITINYLATION_PROTEINS	37	0,60	1,74	0,008	0,015
REACTOME_GLOBAL_GENOME_NUCLEOTIDE_EXCISION_REPAIR_GG_NER	76	0,59	1,74	0,006	0,015
REACTOME_REGULATION_OF_GLUCOKINASE_BY_GLUCOKINASE_REGULATORY_PROTEIN	30	0,65	1,74	0,002	0,016
REACTOME_TRANSLESION_SYNTHESIS_BY_POLH	18	0,74	1,74	0,008	0,015
REACTOME_APC_C_MEDIATED_DEGRADATION_OF_CELL_CYCLE_PROTEINS	76	0,69	1,74	0,002	0,016
REACTOME_COPI_DEPENDENT_GOLGI_TO_ER_RETROGRADE_TRAFFIC	78	0,65	1,74	0,011	0,016
REACTOME_INTERACTIONS_OF_REV_WITH_HOST_CELLULAR_PROTEINS	35	0,65	1,74	0,000	0,015
REACTOME_KSRP_KHSRP_BINDS_AND_DESTABILIZES_MRNA	15	0,68	1,74	0,006	0,016
REACTOME_PIWI_INTERACTING_RNA_PIRNA_BIOGENESIS	21	0,66	1,74	0,006	0,016

© 2020 by the authors. Licensee MDPI, Basel, Switzerland. This article is an open access article distributed under the terms and conditions of the Creative Commons Attribution (CC BY) license (http://creativecommons.org/licenses/by/4.0/).
