# Supplementary material for: Impact of Wnt/β-Catenin Inhibition on Cell Proliferation through CDC25A Downregulation in Soft Tissue Sarcomas
Source: Cancers (Basel). 2020 Sep 8;12(9):2556. doi: 10.3390/cancers12092556 (PMC7564873; doi:10.3390/cancers12092556)

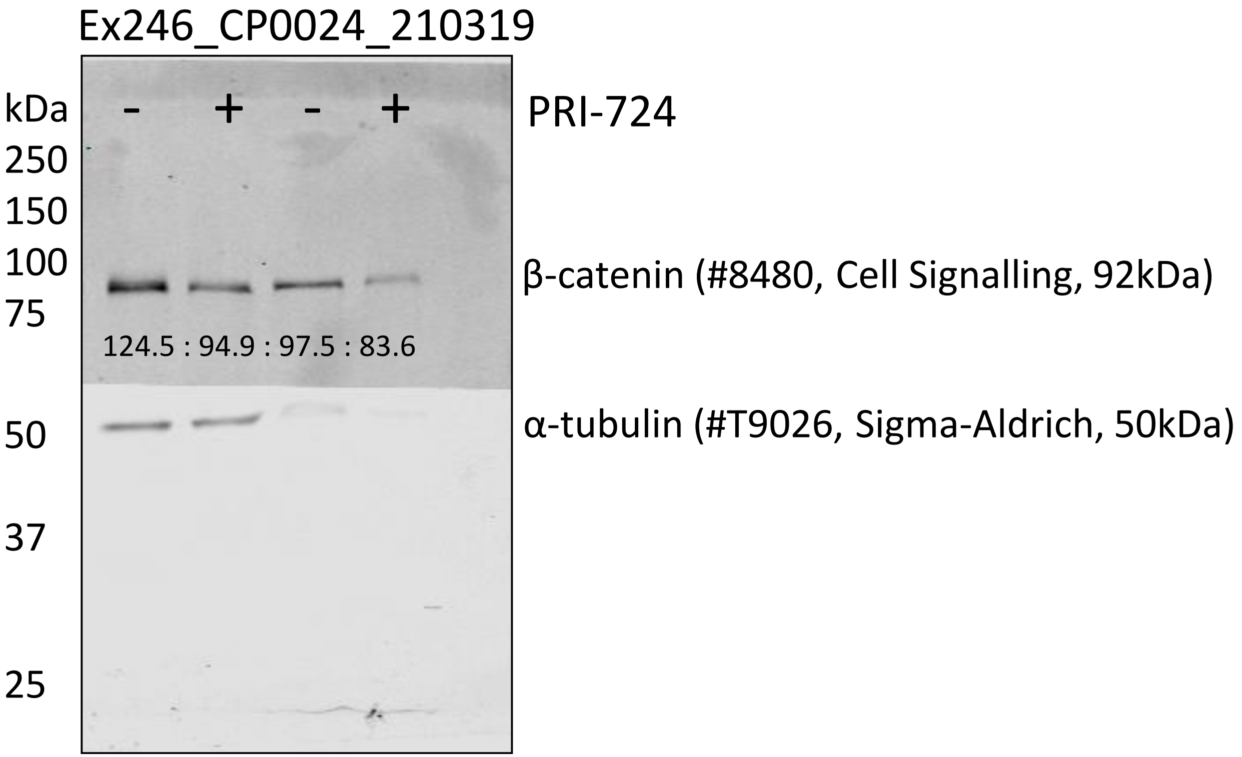
Ex246_CP0024_210319
kDa
250
150
100
75
50
37
25
- + - +
PRI-724
β-catenin (#8480, Cell Signalling, 92kDa)
124.5 : 94.9 : 97.5 : 83.6
α-tubulin (#T9026, Sigma-Aldrich, 50kDa)

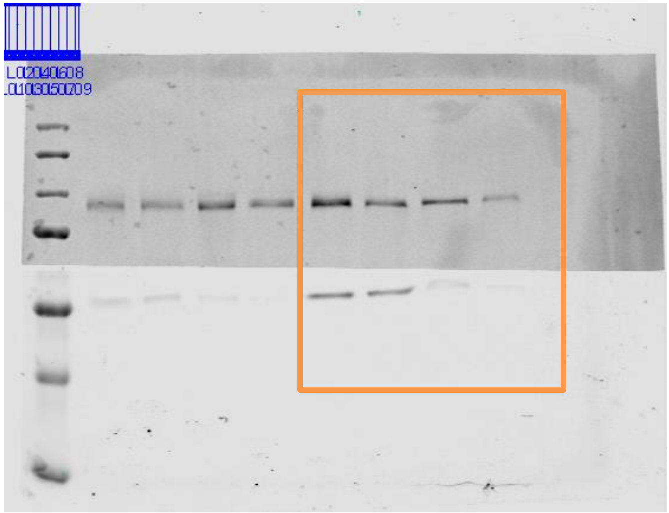

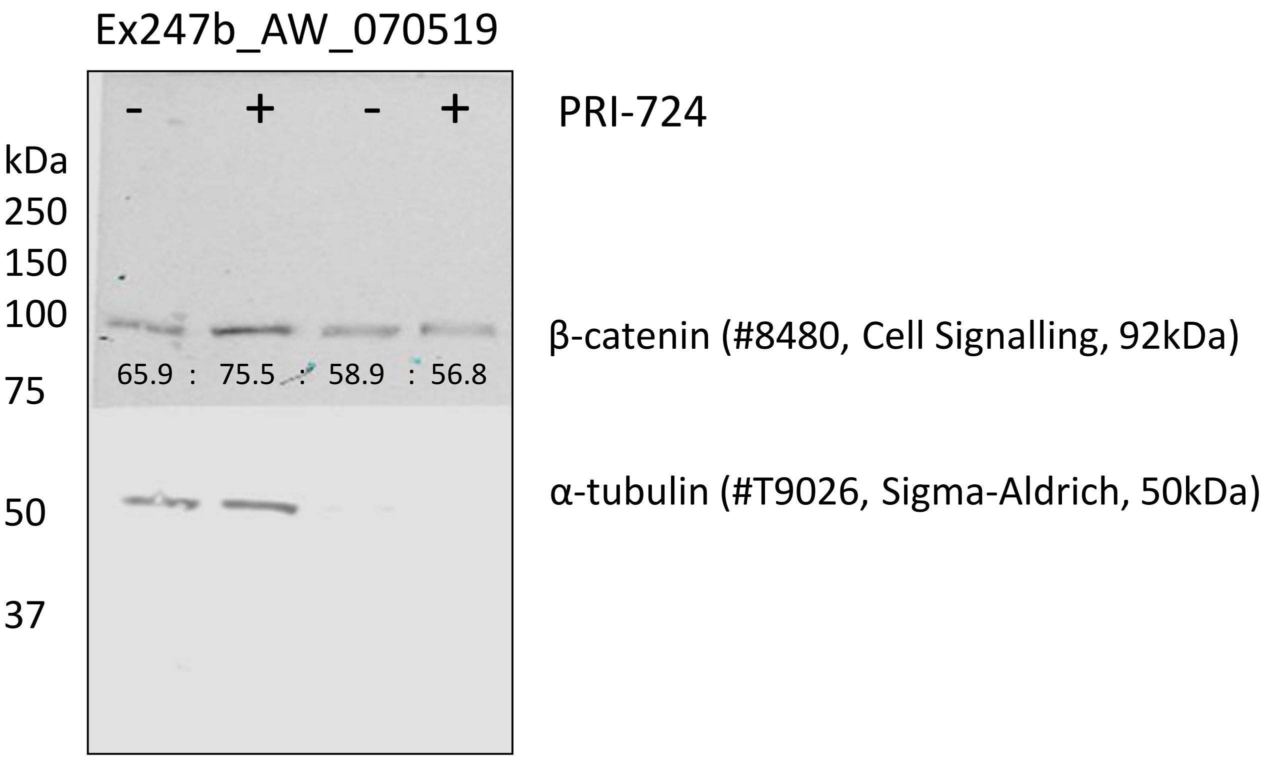

Ex247b_AW_070519
-
+
-
+
PRI-724
kDa
250
150
100
75
50
37
β-catenin (#8480, Cell Signalling, 92kDa)
65.9 : 75.5 : 58.9 : 56.8
α-tubulin (#T9026, Sigma-Aldrich, 50kDa)

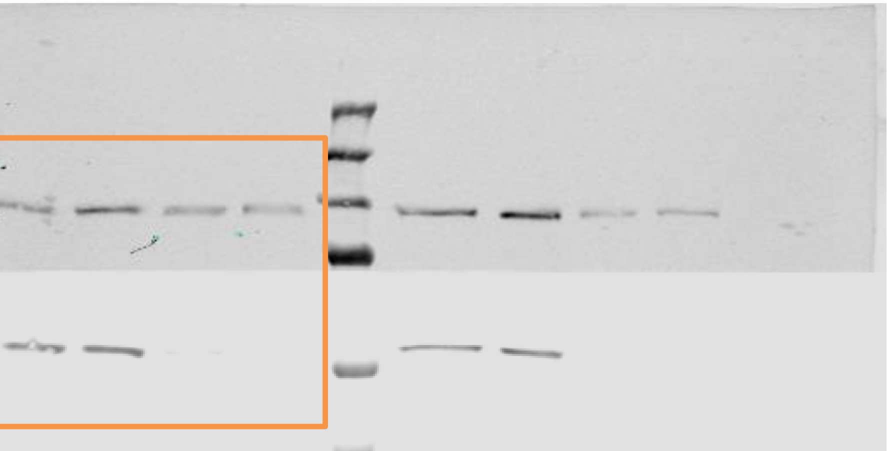

Ex251b_SK-UT-1_070519

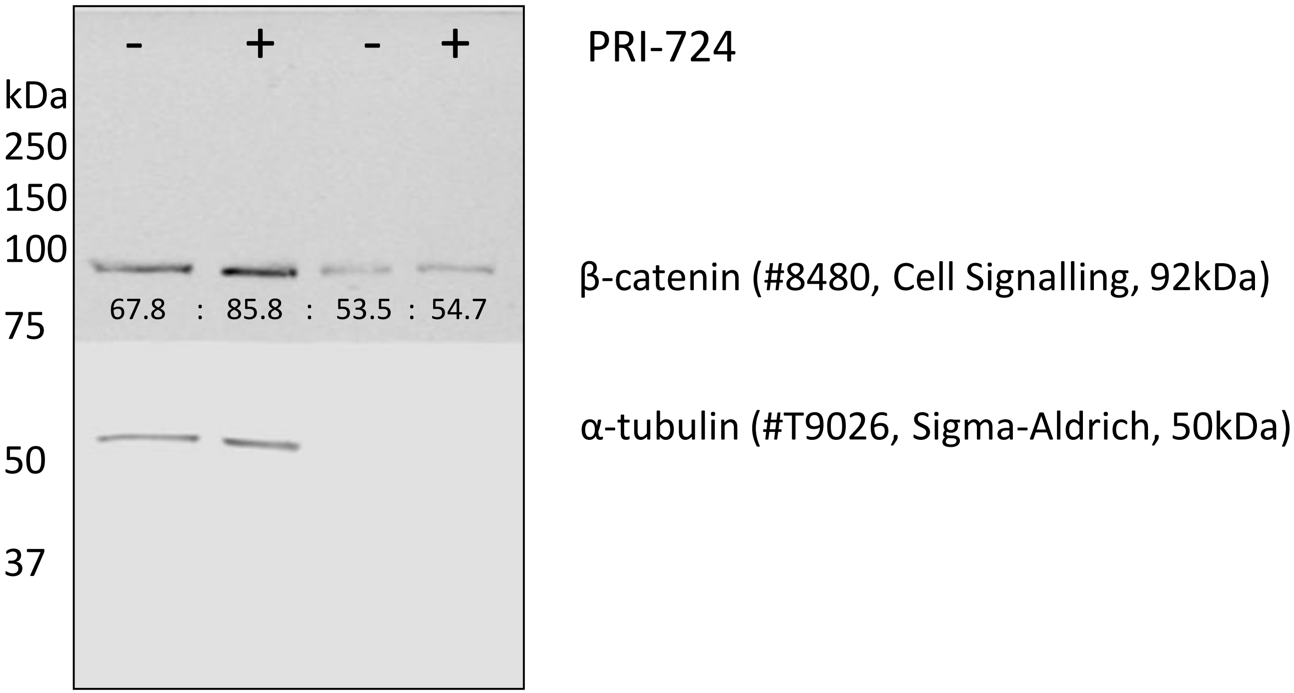

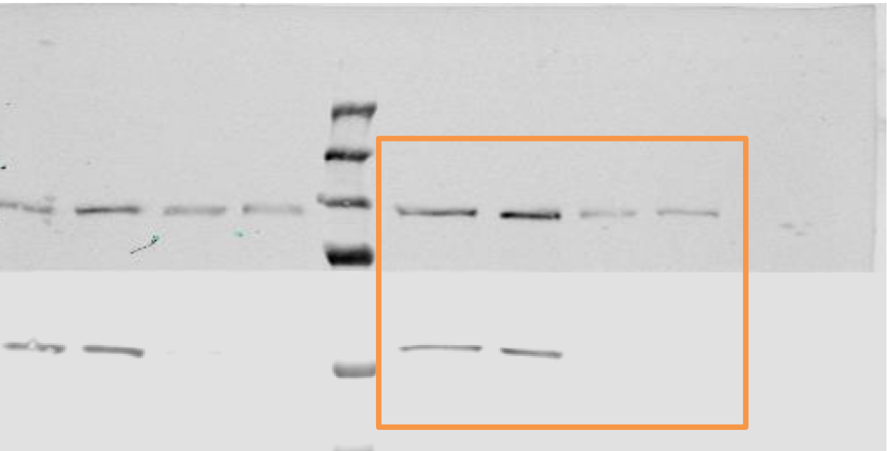

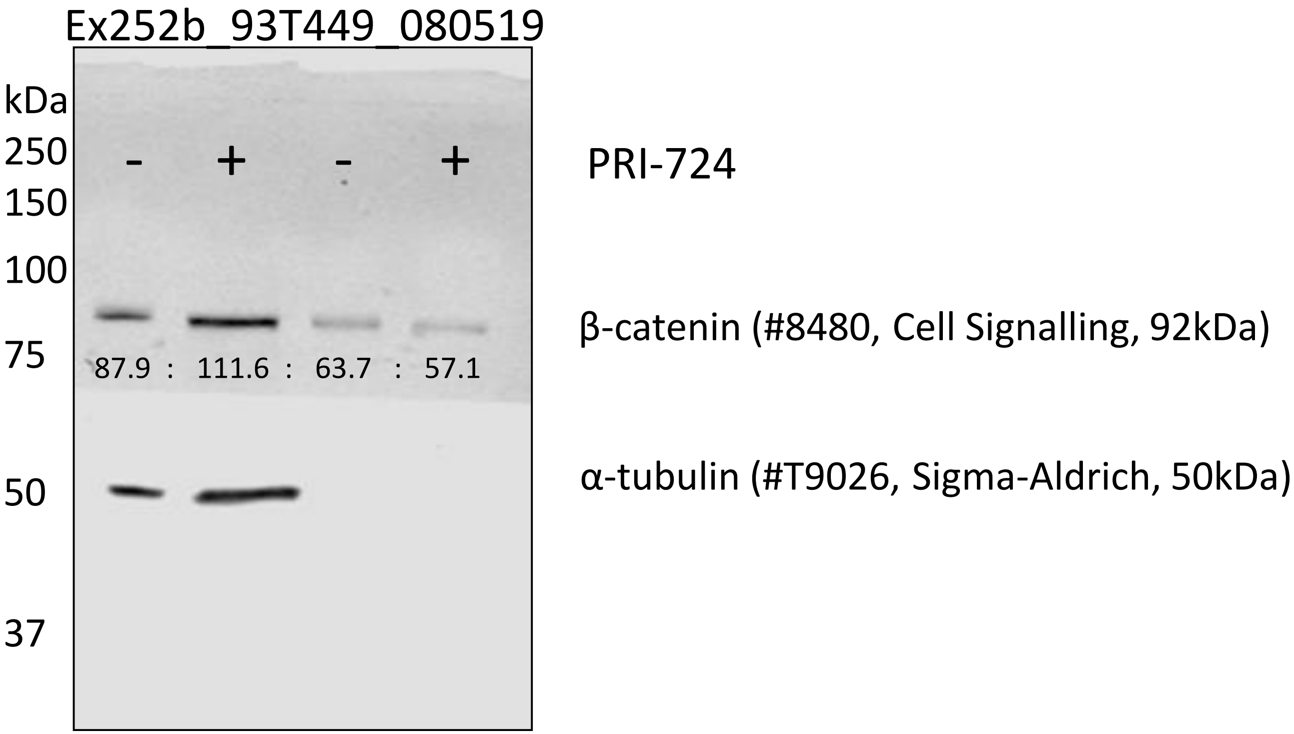
Ex252b_93T449_080519
kDa
250
150
100
75
50
37
- + - +
PRI-724
87.9 : 111.6 : 63.7 : 57.1
β-catenin (#8480, Cell Signalling, 92kDa)
α-tubulin (#T9026, Sigma-Aldrich, 50kDa)

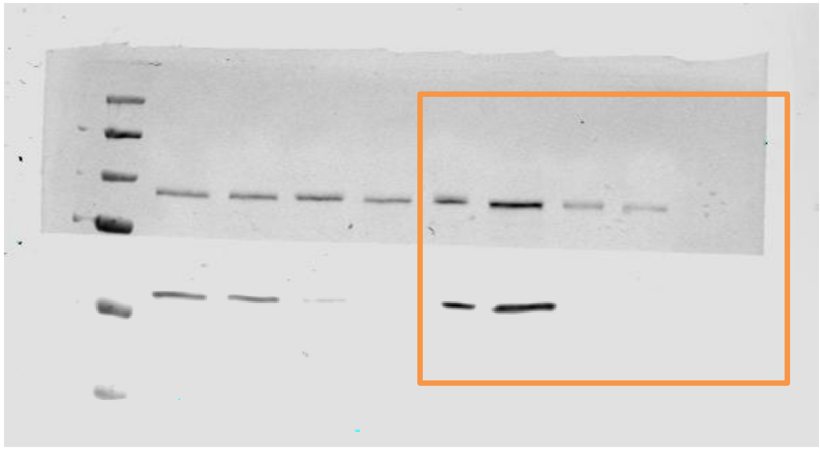

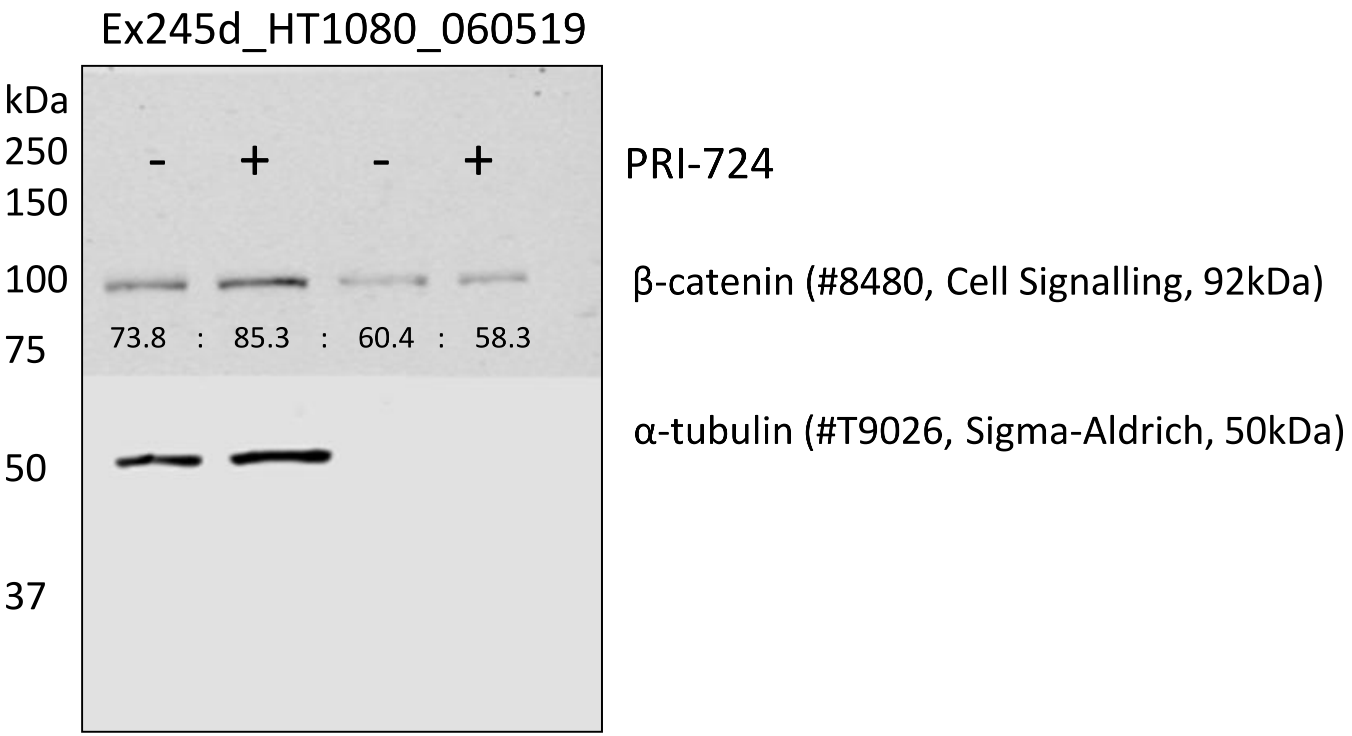
Ex245d_HT1080_060519
kDa
250
150
100
75
50
37
- + - +
PRI-724
β-catenin (#8480, Cell Signalling, 92kDa)
73.8 : 85.3 : 60.4 : 58.3
α-tubulin (#T9026, Sigma-Aldrich, 50kDa)

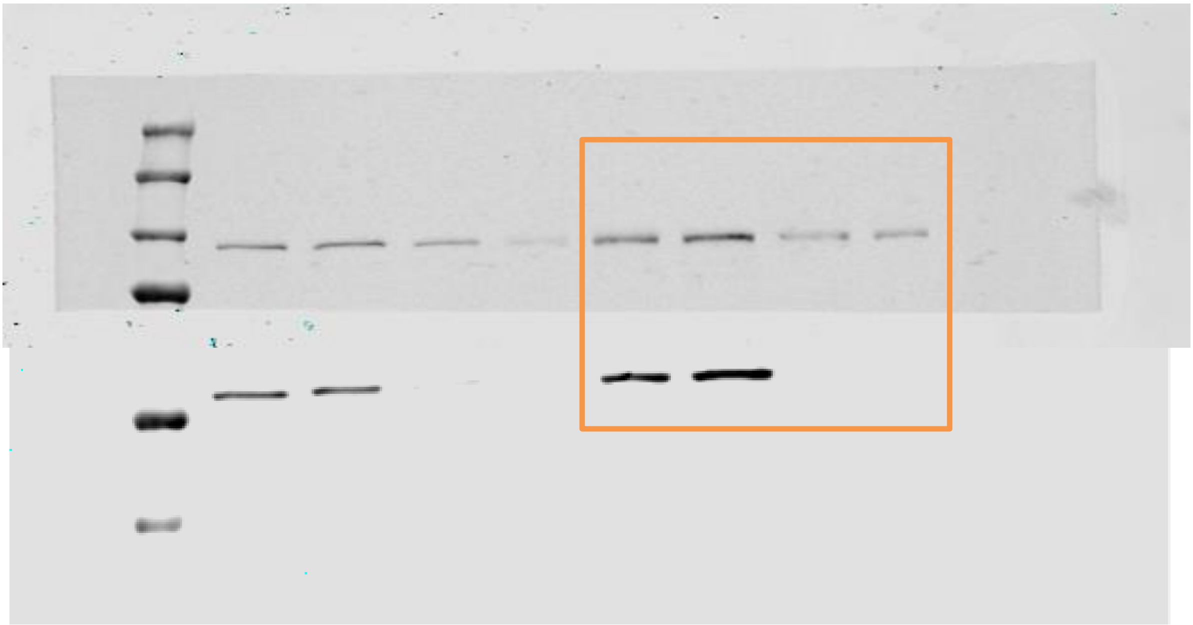

Supplement: Supplementary file 1 [file cancers-12-02556-s001.zip › original WB figures.pdf]
